# The COVID-19 explorer—An integrated, whole patient knowledge model of COVID-19 disease

**DOI:** 10.3389/fmmed.2022.1035215

**Published:** 2022-12-22

**Authors:** Stephan Brock, Theodoros G. Soldatos, David B. Jackson, Francesca Diella, Klaus Hornischer, Anne Schäfer, Simon P. Hoerstrup, Maximilian Y. Emmert

**Affiliations:** ^1^ Molecular Health GmbH, Heidelberg, Germany; ^2^ SRH Hochscule, University of Applied Science, Heidelberg, Germany; ^3^ Institute for Regenerative Medicine, University of Zurich, Zurich, Switzerland; ^4^ Wyss Zurich, University of Zurich and ETH Zurich, Zurich, Switzerland; ^5^ Department of Cardiothoracic and Vascular Surgery, German Heart Institute Berlin, Berlin, Germany; ^6^ Department of Cardiovascular Surgery, Charité Universitätsmedizin Berlin, Berlin, Germany

**Keywords:** SARS-CoV-2, molecular mechanisms, disease modeling, evidence-based medicine, translational research

## Abstract

Since early 2020 the COVID-19 pandemic has paralyzed the world, resulting in more than half a billion infections and over 6 million deaths within a 28-month period. Knowledge about the disease remains largely disjointed, especially when considering the molecular mechanisms driving the diversity of clinical manifestations and symptoms. Despite the recent availability of vaccines, there remains an urgent need to develop effective treatments for cases of severe disease, especially in the face of novel virus variants. The complexity of the situation is exacerbated by the emergence of COVID-19 as a complex and multifaceted systemic disease affecting independent tissues and organs throughout the body. The development of effective treatment strategies is therefore predicated on an integrated understanding of the underlying disease mechanisms and their potentially causative link to the diversity of observed clinical phenotypes. To address this need, we utilized a computational technology (the Dataome platform) to build an integrated clinico-molecular view on the most important COVID-19 clinical phenotypes. Our results provide the first integrated, whole-patient model of COVID-19 symptomatology that connects the molecular lifecycle of SARS-CoV-2 with microvesicle-mediated intercellular communication and the contact activation and kallikrein-kinin systems. The model not only explains the clinical pleiotropy of COVID-19, but also provides an evidence-driven framework for drug development/repurposing and the identification of critical risk factors. The associated knowledge is provided in the form of the open source COVID-19 Explorer (https://covid19.molecularhealth.com), enabling the global community to explore and analyze the key molecular features of systemic COVID-19 and associated implications for research priorities and therapeutic strategies. Our work suggests that knowledge modeling solutions may offer important utility in expediting the global response to future health emergencies.

## 1 Introduction

If there is any positive to be gleaned from the devastating COVID-19 pandemic, it could be the scale and impact of global response from the biomedical research community to the study of the SARS-CoV-2 virus. From efforts to characterize molecular disease mechanisms in the search for tractable therapeutic avenues, to drug repurposing and pandemic forecasting, we have witnessed unprecedented levels of multidisciplinary collaboration. Nevertheless, the resultant peer-reviewed insights have come at a rapid velocity and volume that makes it challenging to efficiently capture, integrate and analyze emergent data from both the clinical and molecular domains. The integration of such insights with existing knowledge is pivotal to efficient knowledge transfer and assimilation by the research community, aiding our understanding of disease mechanisms and expediting the generation and testing of associated therapeutic hypotheses. Recognizing the global urgency, we initiated a COVID-19 focused knowledge modeling effort in March 2020 that sought to rapidly address this challenge. Our goal was to develop a whole patient knowledge model of COVID-19 symptomatology and associated molecular knowledge that links the key molecular players in disease pathophysiology to: 1) common symptoms, 2) severe manifestations and 3) outcome and severity-associated risk factors.

**FIGURE 1:**
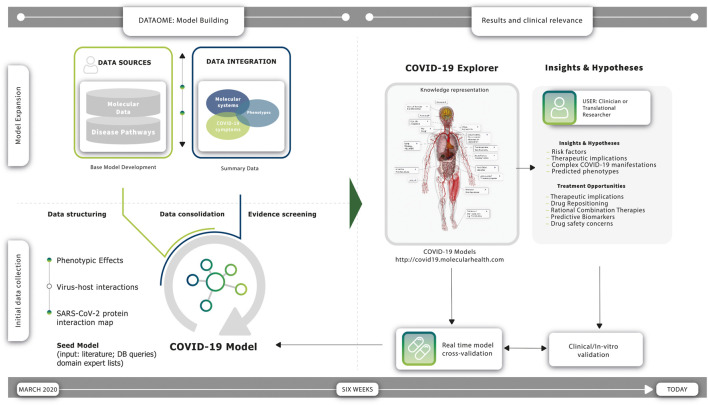
Construction and utility of the COVID-19 molecular symptomology model. Timeline **(*left*)**: Expert-supervised knowledge modeling strategy enabled the rapid construction of a comprehensive COVID-19 Model using Molecular Health’s (MH) Dataome technology. ***Timeline* (*right*)**: a constant flow of new observations during the course of the pandemic has provided real-time validations of the resulting model—several findings are today clinically validated and incorporated in multicenter studies. ***Initial Data Collection*** (***left; bottom***) ***:*** a “Seed Model” was constructed based on information derived from the scientific literature available at the early stage of the pandemic, accompanied by supportive data provided by domain experts and relevant knowledge stored in databases. More specifically, the Dataome knowledgebase of clinical and molecular knowledge was queried using a pre-established SARS-CoV-2 protein interaction map of 332 high confidence human protein interactors within the human proteome (Gordon et al., 2020), involving molecular determinants of host cells and the differential impact of the viral infection on the host cell response. ***Model Expansion*** (***left; upper***) ***:*** the ‘Seed Model’ was normalized, computationally structured and automatically connected to broader biomolecular information to provide the “Base Model”—using Dataome core data integration technology (see also Figure 3), the “Base Model” was iteratively expanded further *via* an expert-guided strategy. This computer augmented evidence prioritization effort revealed a set of converging central molecular pathophysiology mechanisms. ***Results*** (***right***): findings were organized in three different representations providing overlapping and cross-connected functional utilities for the interested clinician and/or translational researcher, namely a network-based view of the COVID-19 Model encompassing a higher-level summary of the eight key pathogenic mechanisms found to be associated with COVID-19 clinical phenotypes (see also Figure 4), a web-based ‘COVID-19 Explorer’ that links each component of the interconnected COVID-19 Disease Model network to underlying views with additional information and detailed evidence summaries (publicly available at http://covid19.molecularhealth.com), and the “COVID-19 Cockpit”—a guide summarizing the COVID-19 Model in a tabular format with human understandable texts explaining per row reported COVID-19 clinical phenotypes, associated pathogenic mechanisms, and their relation to the COVID-19 Model with respect to each of the eight key SARS-CoV-2 perturbed mechanisms (including links to the respective ‘COVID-19 Explorer’ description-views). ***Clinical significance*** (***right***): the COVID-19 Model and its representations enable direct clinical or therapeutic support by providing both causal and inferred insights that reflect the symptomology of patients and their individual characteristics (see Supplementary Table S1)—importantly, continuous synchronization with new SARS-CoV-2 data and MH Dataome content is warranted for quick-response, and fast informed data and hypothesis generation towards improved COVID-19 management and better understanding of its multifaceted phenotype manifestations and duration. Treatment modalities indicated by the model include drugs associated with modulation of *ACE2* induction, *B1R* agonists, as well as *Kallikrein* inhibitors, *SerpinG1* enzyme replacement and *SIRT-1* or *PPARγ* activators. Several of these are currently investigated in late phase COVID-19 clinical trial studies (see also (Brock et al., 2022)).

To achieve this, we designed a stepwise, expert-driven knowledge modeling strategy that iteratively combined the capacities of both extensive data integration and human insight (see Figure 1). This supervised strategy helped us to 1) manage the rapid pace of new insights, 2) enable the flexible elaboration of more specific disease symptom models and associated hypotheses, and 3) permit the real-time inclusion of important new findings at the whole-patient level. Here, we report on our findings and the functionality of the COVID-19 Explorer web resource. Our results are provided at the level of a whole-patient knowledge model, including the possible causative pathogenic mechanisms underlying COVID-19 phenotypes (Brock et al., 2022). To accommodate usability for a variety of use-case scenarios, results are summarized in different formats: the ‘COVID-19 Explorer’ provides a detailed, comprehensive and fully interactive view of the relationships and accompanying evidence, while ‘The COVID-19 Cockpit’ (see Supplementary Table S1) is intended to support clinical researchers in diagnostic and hypothesis generation for new therapeutic strategies (see Figure 1).

By developing a patient-level molecular atlas of COVID-19 pathogenesis as an interactive open-source knowledge model, we provide the biomedical and clinical research communities with an effective tool to decipher COVID-19 hypotheses and to enable more informed development and testing of new diagnostic and therapeutic strategies.

### 1.1 Materials and methods

**FIGURE 2:**
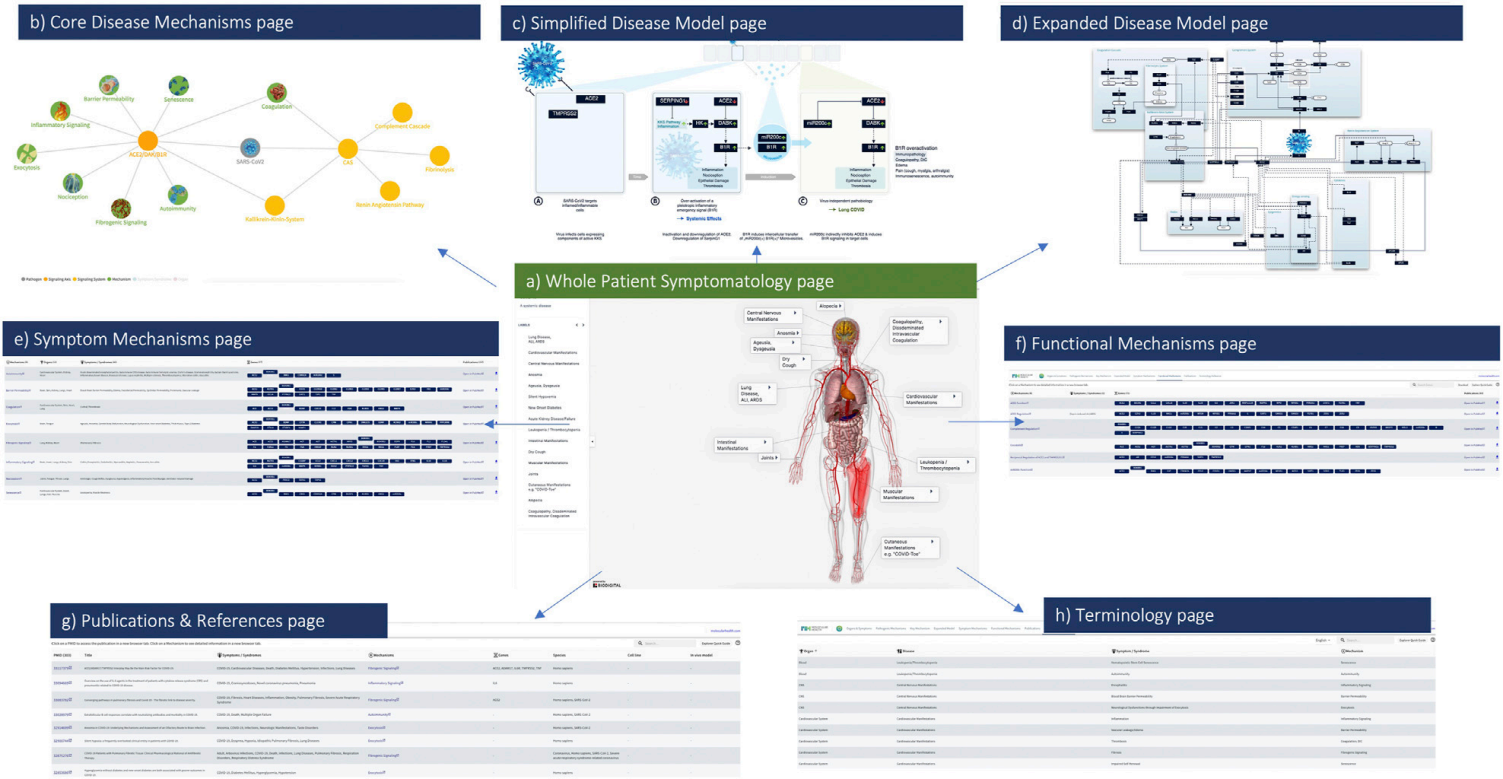
The COVID-19 Explorer Interface. Screenshots from the COVID-19 Explorer highlighting how users can navigate through different levels of detailed visualizations, annotated graphics and reference information. Key pages within the interface include **(A)** Whole patient symptomatology **(B)** eight converging pathogenic disease mechanisms **(C)** a simplified disease model **(D)** an expanded disease model **(E)** symptom mechanisms **(F)** functional mechanisms **(G)** publications and **(H)** terminology references. Each of these views is designed to assist translational researchers in both hypothesis development and testing for COVID-19.

We utilized Molecular Health’s (MH’s) Dataome technology platform in collaboration with disease modeling experts to capture, structure and logically connect diverse clinical and molecular features of COVID-19 pathobiology (see Figure 1). Readouts from these rapid *in silico* analyses were curated and organized into molecular models containing salient information for each symptom (see Figure 2 and Figure 4). The final complete comprehensive model, linking key molecular mechanisms to COVID-19 symptomatology and the related source data, is made publicly available *via* the web-based COVID-19 Explorer (accessible, at http://covid19.molecularhealth.com).

#### 1.1.1 The Dataome technology

Our studies utilized the Dataome technology as the core data integration, knowledgebase and analytical framework. Dataome was designed to enable the constant capture and curation of globally available data sources of clinical and molecular knowledge, with the aim of delivering quality controlled data for clinical decision-making and knowledge discovery in a disease agnostic manner. It consists of three core components (see Supplementary Material S1, Figure 1):

• Dataome Capture—uses an ensemble of public/proprietary algorithms and resources to enable the global harvesting, quality assurance and integration of emergent clinical and molecular data. Structured data is assimilated using automated data integration pipelines that process, normalize and quality assure the data in synchrony with database update cycles. These functions utilize an extensive infrastructure that enables extraction, transformation, and loading (ETL) of source data into a consolidated database framework for information modelling and knowledge extraction. For unstructured data, text and data mining (TDM) technologies provide an ensemble of natural language processing (NLP) functions, such as rule-based linguistic, machine learning and deep learning models, trained to identify critical biomedical terms and relationships from any source of unstructured knowledge (e.g., drug labels, patents and peer-reviewed literature). This machine-reading framework enables targeted extraction of biomedical facts that are fed to a proprietary curation infrastructure for review by biomedical experts. Curated and quality-controlled data is then integrated into the Dataome’s Nucleus.
• Dataome Nucleus—a comprehensive data and knowledge resource containing highly interconnected clinical and molecular data, linking clinical phenotypes to underlying molecular knowledge. Nucleus encompasses data from more than one hundred (100+) public and commercial/private resources, including in-house proprietary databases (see Figure 2 in Supplementary Material S1). Public datasets span a broad range of content, size and formats—from more general, such as literature [e.g., PubMed (Sayers et al., 2021; Kim et al., 2021)], biomedical ontologies [e.g., ICD (Krawczyk et al., 2020), MedDRA (Brown, 2004), ATC (Merabti et al., 2011), MeSH (Lipscomb, 2000), UMLS (Humphreys et al., 2020)] or information about proteins and genes [e.g., from resources like UniProt (UniProt Consortium, 2021), Entrez Gene (Maglott et al., 2011), Ensembl (Howe et al., 2021), UCSC (Gonzalez et al., 2021), or RefSeq (O'Leary et al., 2016)], to more targeted information, such as genomic variant annotations [e.g., ClinVar (Landrum et al., 2020), dbSNP/dbNFSP (Sayers et al., 2021)], information about drugs and their labels, targets or interactors [e.g., DrugBank (Wishart et al., 2018), FDA Orange Book (Ursu et al., 2018) or Drugs@FDA (Ursu et al., 2018)], or biomolecular pathways and interactions [e.g., KEGG (Kanehisa et al., 2021) and Reactome (Jassal et al., 2020)]. Other resources include real-world data (RWD) such as public pharmacovigilance repositories [e.g., VigiBase (Fernandez et al., 2020) or FAERS (Yao et al., 2020)], as well as information regarding clinical trials (e.g., from NCT’s clinicaltrials.gov). The system also contains structured information regarding therapeutic guidelines and variant classification [e.g., ACMG (Harrison et al., 2019), NCCN (Koh et al., 2020) or ESMO (Cherny et al., 2017)], as well as further curated datasets pertaining clinical biomarker interpretation, pathway/interaction relationships, drug and clinical trial information.
• Dataome Analytics—provides a portfolio of analytical solutions designed to derive new insights from the data contained in Nucleus. This data provides the evidence-base to support the development of both commercial decision support technologies [e.g., MH EFFECT (Schotland et al., 2021)].
• and MH GUIDE (Hirotsu et al., 2020) and the efficient development of disease models for any human disease or phenotype, in this case COVID-19. These software tools are complemented by specialized analytical pipelines that integrate bioinformatics, chemoinformatics, systems biology, clinical data science, and AI/machine learning (e.g., with integrated analysis, feature engineering and powerful pre-trained models) based methodologies.

The utility of the integrated Dataome technology has been previously validated across multiple clinically important contexts, including biomarker discovery, drug safety prediction and drug repositioning (Armaiz-Pena et al., 2013; Bohnert et al., 2017; Pradeep et al., 2015; Soldatos et al., 2018; Soldatos and Jackson, 2019; Schotland et al., 2021; Schell et al., 2016). While Dataome provides a flexible approach to the automated capture and quality assurance of globally available sources of clinical and molecular data and knowledge, a supervised approach was required for the molecular modeling of COVID-19 to account for the rapid emergence of new insights. This permitted the stepwise, expert-guided elaboration of more specific COVID-19 symptomology models and associated hypotheses, flexible enough to enable the near real-time inclusion of important new findings, given the highly dynamic nature of the pandemic.

### 1.2 COVID-19 knowledge modeling strategy

#### 1.2.1 “Base model” generation

To initiate the model building process, we queried the Dataome Nucleus with the previously reported SARS-CoV-2 protein interaction map of 332 high confidence interactors (Gordon et al., 2020) as a “seed” for knowledge expansion. To optimize the specificity of our analysis, we focused on the molecular determinants of host cells that define them as viral targets and the immediate impact of the viral infection on the host cell response. This was achieved through inclusion of a minimum set of elements defining host cell, host-specific response (e.g., innate immune response) and associated phenotypes (see Figure 1). Domain experts independently inspected resultant data, providing a systematic expansion of the network to include related pathways, protein interactors, and regulatory elements. This so-called “base model” centered on a converging molecular mechanism including the host proteins responsible for virus entry, TMPRSS2 and ACE2, together with significantly differentially down-regulated components of the interferon stimulated genes (ISG) induced by the virus infection (ACE2 and SERPING1) (see Figure 1).

#### 1.2.2 Iterative expansion of the base model

The base model was further expanded through integration of key molecular protagonists associated with COVID-19 pathophysiology and symptomatology (see Figure 1) including:

i) Common disease symptoms (e.g., dry cough, myalgia, anosmia, dysgeusia/ageusia, metallic taste sensation, thick mucus, transient diabetes, silent hypoxia, leukocytopenia, and central nervous system (CNS) manifestations).
ii) Severe manifestations (e.g., Acute Respiratory Distress Syndrome (ARDS), acute lung infection (ALI), lung fibrosis, cardiovascular complications including arrhythmia and acute coronary syndrome (ACS).
iii) Outcome and severity associated risk factors (e.g., age, sex, smoking, air pollution, comorbidities).

**FIGURE 3:**
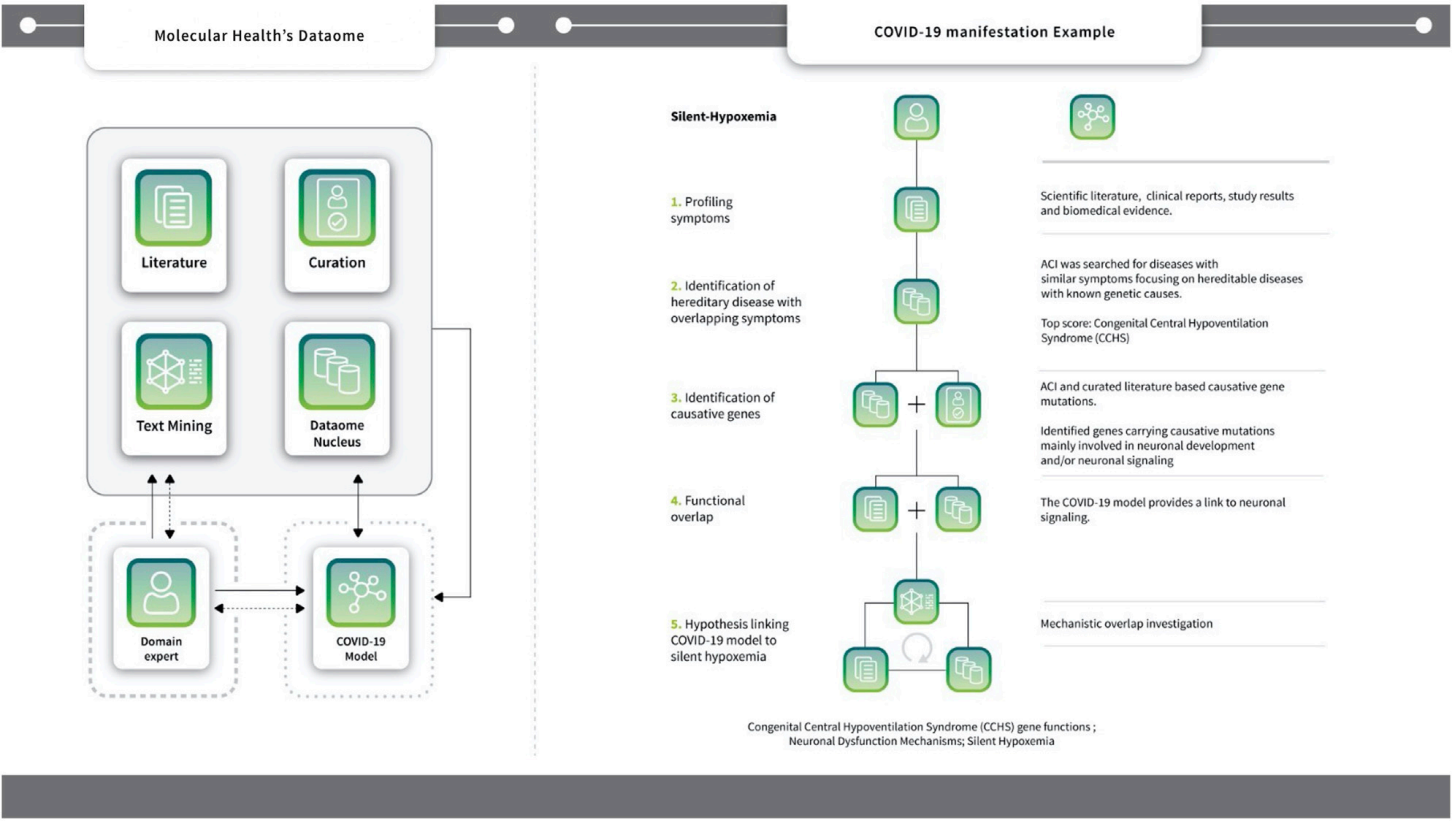
Symptom Analysis Workflow using the case-study example of Silent hypoxemia. The Dataome knowledgebase provides an extensive database (DB) infrastructure that integrates more than 100 biological, chemical and clinical resources; see also Supplementary Figure S1. It also includes analytics for accessing and analyzing life science data and literature (i.e. text-mining and curation functionalities - see central single-line box). Dataome can be used stand-alone or to support the activities of human domain experts (see, long dotted-line box). Here, the COVID-19 model was built following a computer augmented approach that integrated a DB-driven strategy with iterative expert knowledge and manual input. Non-dashed arrows, indicate the main “flow of action” towards the goal of producing the COVID-19 model (see, short dotted-line box). Experts provided input or queried Dataome to construct key model aspects, but also processed the model directly (e.g., during representation preparation or when investigating sub-networks). Data from the model were also used as input to query Dataome and, in turn, to update/extend the original model. Dashed arrows indicate additional data flow/actions that were not main or central activities - namely, when domain experts received feedback from the Dataome and/or the COVID-19 model to augment it with new data or proceed with next steps and/or analysis (***Right***) One such example of COVID-19 hypothesis generation that combined manual domain expertise and multiple components of MH’s Dataome relates to silent hypoxemia: the diagram illustrates the five key steps towards the identification of functional link with Congenital Central Hypoventilation Syndrome (CCHS) genes *via* neuronal signaling/dysfunction mechanisms. Another example of such a hypothesis generation workflow - this time, based on four steps - *B1R* mediated sensitization of *TRPV4* signaling to the incidence of ventilator induced acute lung injury (VILI) in COVID-19 patients is found in Supplementary Figure S3).

Specific symptomatology associated ‘pre-models’ were then manually defined, driven by the biomedical domain experts who inspected and curated the clinico-molecular data extracted previously *via* the interrogation of the Dataome knowledgebase (see examples in Figure 3). The expert-driven process to elucidate the molecular underpinnings of COVID-19 and the diversity of associated disease phenotypes and risk factors focused on three key goals:

• Unravel the molecular foundations of the systemically observed symptoms
• Assess whether a core innate immune response might explain the multiple post-infection reactions, and
• Identify risk factors and phenotypes towards prioritization of drug candidates.

#### 1.2.3 Pre-model curation and integrated visualization

At each stage of the disease/symptom modeling process, the extracted “pre-models” were inspected and visually modeled using the free open-source software PathVisio, [version 3.2.2 (Kutmon et al., 2015)]. During this process, relevant “pre-model” references were attached to the respective objects in PathVisio and manually complemented with bibliography as necessary. Entities and relationships associated with each mechanism/symptom were presented by a JavaScript-animated SVG image. Graphical renditions were produced *via* the PathVisio program, with data outputs in GPML and SVG format.

### 1.3 The web-based knowledge explorer

To enable the effective exploration of the key findings by the community regarding the mechanisms that possibly underlie COVID-19 symptoms and the associated evidence summaries, we developed a dedicated web-based interface providing a comprehensive and fully interactive COVID-19 disease model. Results are also provided in the format of a printable Table (see Supplementary Table S1) An expanded version of this Table is found in the sister article to this one focusing in detail on the molecular hypotheses and their validation status1.

#### 1.3.1 Whole patient pre-model integration

**FIGURE 4:**
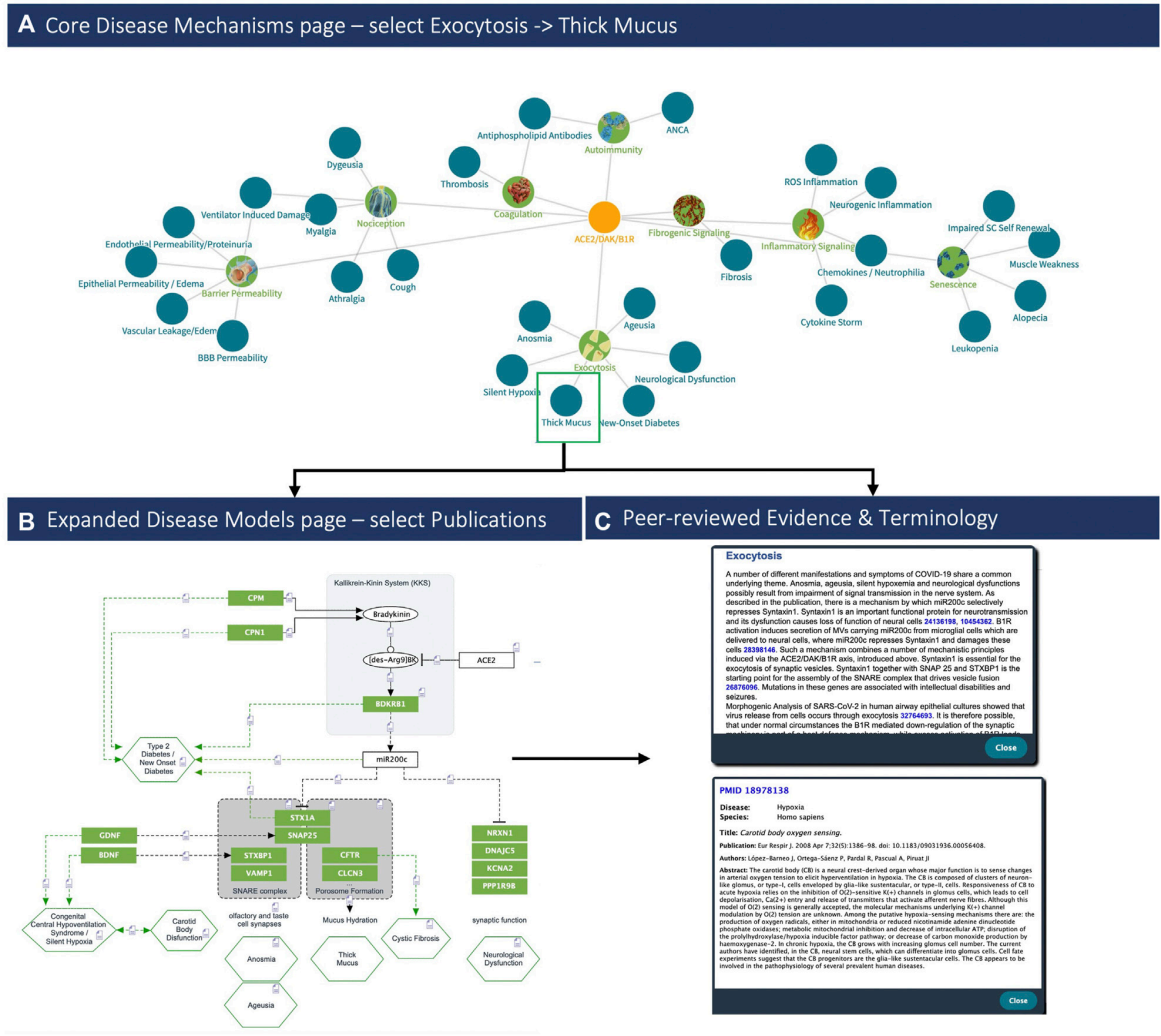
COVID-19 clinical phenotypes associated with eight key pathogenic mechanisms (*Left, up*) Example of the **(A)** *Disease Mechanisms* tab: showing the key mechanisms (green nodes) and associated signs, symptoms and syndromes of COVID-19 (blue nodes) induced by over-activation of the *ACE2/DAK/B1R* signaling axis. Members of the axis, as well as regulatory elements (e.g., miR200c, *SIRT1*, *EZ*H2, etc.) converge at these mechanisms and complement each other to various degrees in inducing the corresponding phenotypes (Top). Clicking on the ‘Exocytosis’ node of the *Disease Mechanisms* tab leads the user to **(B)** the respective sub-model pathway viewer that summarizes associated components, mechanisms and pathogenicity symptoms (bottom, left). Nodes may be highlighted in green if they indicate molecular players related to symptoms or in blue if the respective components relate to the virus infection. Finally, **(C)** each sub-model is described also in free text summaries. For each edge, associated literature is optionally available, too (bottom right).

**FIGURE 5:**
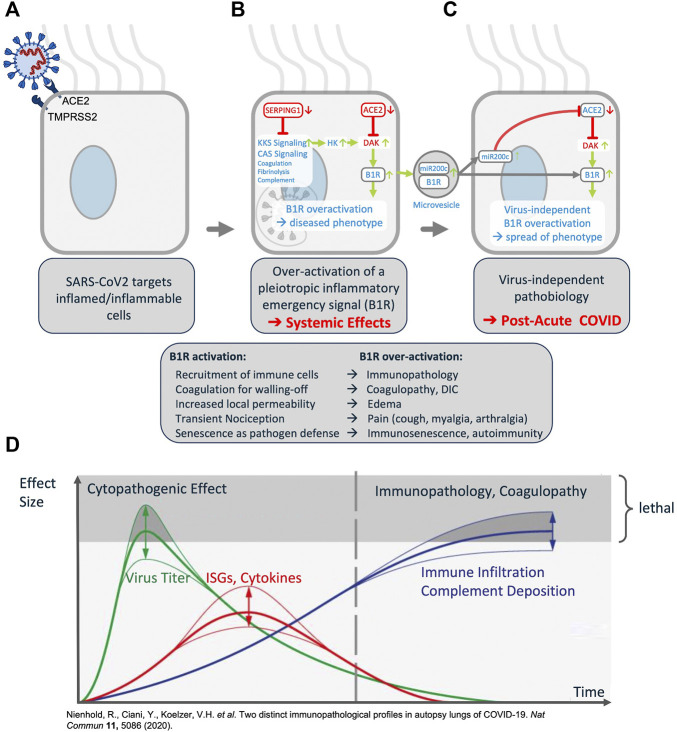
Synopsis of the COVID-19 Disease Model. **(A)** The virus targets host cells expressing *ACE2* and *TMPRSS2*, active components of the KKS. **(B)** Within the cell the virus induces downregulation of *SERPING1* and *ACE2*. *SERPING1* downregulation induces activation of the CAS and KKS. *ACE2* downregulation results in accumulation of *DAKs*. Excess *DAKs* activate *B1R*, triggering constitutive activation and auto-amplification. Under normal conditions *B1R* activation triggers a fast, transient inflammatory emergency reaction inducing neutrophil and leukocyte recruitment and infiltration, opening of the epithelial/endothelial barriers for their transmigration, coagulation for local walling-off, fibrogenesis for wound healing, senescence as host defense mechanism and transient nociception. Constitutive activation of this system leads to excess inflammatory signaling, epithelial/endothelial barrier breakdown, induction of thrombosis, fibrosis, pain and other effects. **(C)** *B1R* signaling induces the formation of MVs bearing *B1R* and miR200c. These are transferred to target cells, inducing further expression of miR200c, which leads to *ACE2* downregulation and formation of excess *DAKs* which signal *via B1R*. Thus, MV transfer *via* auto-activation and amplification of the *B1R* system may trigger the virus independent propagation of an inflammatory phenotype. **(D)** Schematic time course of COVID-19 lung disease based on lung autopsy findings (illustration adapted with permission from (Nienhold et al., 2020)). This time course is consistent with the proposed model: early in the disease, an ISGhigh lung profile is observed, with high viral load, high expression of cytokines and ISGs, and sparse immune infiltrates. Late in the disease, an ISGlow lung profile is observed, with low viral load, low local expression of cytokines and ISGs, and strong infiltration of macrophages and lymphocytes. Patients who die early are not able to adequately control SARS-CoV-2, while patients who die late suffer from coagulopathy and immunopathology. This indicates that at a later stage the disease becomes virus independent.

Linkage of the curated pre-models through related molecular protagonists resulted in a whole patient COVID-19 disease model that connects central molecular disease mechanisms (namely, aberrant contact activation system (CAS) and ACE2/DAK/B1R signaling) (see Figures 4, 5) to eight core pathogenic processes: 1) inflammatory signaling, 2) coagulation, 3) barrier permeability, 4) senescence, 5) autoimmunity, 6) fibrogenic signaling, 7) nociception and 8) exocytosis. The model is completed by functionally intersecting these mechanisms with respective symptoms, associated pathogenic pathways and affected organ-systems (see Figure 4 and Supplementary Table S1). The dynamic nature of the global developments around the pandemic provided a constant flow of updated observations that were used as real-time validation of the resulting model’s core1. Finally, domain expert curation ensured continuous synchronization with new SARS-CoV-2 data and Dataome content.

#### 1.3.2 Webserver modules

A Flask micro web framework serves as the web application displaying the interconnected “sub-models”. The application presents an SVG-based graphic for each sub-model, animated by the d3. js JavaScript library. To project the most actual and comprehensive information, the collection of citations supporting the relations of each “sub-model”, may range from peer-reviewed articles to very recent conference content (e.g., abstracts) and even ad-hoc communications. This information is provided as auxiliary information *via* respective animated components.

### 1.4 Results

Constructed during the early stages of the COVID-19 pandemic in 2020, our COVID-19 knowledge model revealed that the multitude and complexity of observed, and seemingly disparate clinical phenotypes may be linked to the pleiotropic activity of eight core molecular mechanisms involved in the host response (see Supplementary Table S1 and 1). In addition, the model revealed functionally connected mechanisms across multiple organ systems allowing for the identification of novel hypotheses for both viral dependent and independent disease mechanisms and associated pharmacologic targets that may warrant further investigation for drug repurposing and/or development efforts1.

#### 1.4.1 The COVID-19 explorer

The COVID-19 Explorer represents a comprehensive COVID-19 disease model linking curated molecular protagonists at the symptom specific level. The COVID-19 Explorer is openly available *via* an interactive web-based interface at: https://covid19.molecularhealth.com.

##### 1.4.1.1 Organization and functionality

The interactive interface provides summarized views between molecular mechanisms and disease processes linked with respective symptoms, associated pathogenic pathways and affected organ-systems. More specifically, the user interface consists of eight components that allow users to explore a number of detailed visualizations and annotated graphics (see Figure 2):

• Organs & Symptoms: consists of a three-dimensional human model that graphically summarizes the major organs and symptoms associated with COVID-19—the model contains hyperlinks leading to the respective underlying biomedical models.
• Pathogenic Mechanisms: contains an interactive network diagram summarizing connections between COVID-19 symptoms, affected organs, functional mechanisms, and key signaling axes—nodes are hyperlinked to the respective views with the details of each model.
• Disease Model: a schematic synopsis of the central mechanisms identified pertaining to cell damage, even in cells not directly infected by the SARS-CoV-2 virus.
• Expanded Model: visual view of pathways, molecular mechanisms, and biological systems affected by the virus—nodes hyperlink to lists of respective components involved (whether listed under Symptom or Functional Mechanisms, or both).
• Symptom Mechanisms: list of components connected to each molecular model (symptoms, organs, associated genes and proteins, references).
• Functional Mechanisms: list of mechanisms triggered by the SARS-CoV-2 virus, and the related genes and proteins.
• Publications: list of hundreds of citations underlying the COVID-19 Explorer—each reference contains hyperlinks to the respective model page and PubMed record.
• Terminology Reference: organ, disease, symptom and mechanism term groupings as considered for the purpose of this work and interface.

These different views are aimed at enabling multiple use case scenarios, relevant to both the research community and clinical users. The full scope of “COVID-19 Explorer” features and utilities is described in detail within the accompanying User documentation: https://covid19.molecularhealth.com/MHCoronaExplorer_QuickGuide.pdf.

##### 1.4.1.2 Using the explorer: Phenotype associations and predictive potential

Our model was developed during the initial stages of the pandemic. At this time, new and seemingly unrelated clinical phenotypes were concurrently described. Using the Explorer, these could be immediately linked to core mechanisms (e.g., barrier permeability or exocytosis). For instance, first reports of silent hypoxemia emerged in April 2020. The symptoms of silent hypoxemia were mapped against the symptomatology of heritable diseases, identifying Congenital Central Hereditary Hypoventilation Syndrome (CCHS) as a phenotypically related disease (see Figure 3) (Brock et al., 2022). CCHS is caused by dysfunction of the exocytosis machinery in oxygen sensing cells, providing a direct link to our model (Supplementary Material S1, Figure 3) (see also Figure 4). A similar link between clinical phenotypes and our model was established upon the first reports on endotheliitis (Varga et al., 2020), vasculitis and the role of micro-thrombotic events in severe disease (see Supplementary Table S1). Here too, direct links between the molecular etiology of the observed symptoms and our model could be drawn and strikingly, by mid-April, first cases of new onset KWD-like disease were reported in children with COVID-19, thereby also suggesting the predictive potential of the model.

##### 1.4.1.3 Key aspects of the COVID-19 knowledge model

The COVID-19 Explorer and associated knowledge model highlight the key molecular players involved in host responsible for SARS-CoV-2 entry (*ACE2*, *TMPRSS2*), and the host factors of the ISG response that are specifically dysregulated by the SARS-CoV host interaction (*ACE2*, *SERPING1*) (see Figure 5). In turn, these are seen to converge on unifying pleiotropic signaling pathways comprising Renin-Angiotensin System (RAS) and Kallikrein Kinin System (KKS) as part of the Contact Activation System (CAS). Concurrent downregulation of *ACE2* and *SERPING1* may then reciprocally amplify the deregulation of KKS thereby generating a `“perfect storm”, which may lead to extreme over-activation of downstream signaling, especially in acute COVID-19. Finally, the model indicates that viral perturbation of eight key mechanisms, alone or in combination, may contribute to the pathogenesis of primary COVID-19 phenotypes.

A full analytical overview of the generated model is summarized in Supplementary Table S1 and made available *via* our COVID-19 Explorer. In addition, extended information about specific mechanisms, pathways, clinical phenotypes and insights into the clinico-molecular hypotheses derived from our model are reported in the associated back-to-back publication (Brock et al., 2022).

##### 1.4.1.4 Host factors mediating SARS-CoV-2 infection

The model identified a converging molecular landscape, delineating host-factor responses to SARS-CoV-2 *via* host proteins responsible for virus entry, as well as significantly differentially down-regulated components of the set of ISG induced by virus infection. More specifically, the model indicates that the cell-entry mechanism (see Figure 5A) and disease-specific ISG signature provides three key active components of SARS-CoV infected cells (*TMPRSS2*, *ACE2* and *SEPRING1*) that may functionally converge in the same pleiotropic signaling systems, namely the CAS and KKS pleiotropic signaling (see Figure 5B).

##### 1.4.1.5 Host-response driven disease mechanisms

The model reveals that SARS-CoV-2 targets cells expressing constituents of a highly inducible inflammatory signaling system causing its excess activation—the pleiotropic nature of this system appears to underpin the diverse clinical manifestations of COVID-19. Importantly, the model indicates also a possible mechanism through which a disease phenotype may be propagated even in the absence of the original viral trigger (see Figure 5C). More specifically:

a) Convergence in pleiotropic KKS may dysregulate the ACE2-DAK-B1R axis, triggering systemic disease (see Figure 5B).
b) Auto-induction of B1R bearing microvesicles (MVs) and interplay with the regulatory miR200c may provide a feed-forward loop decoupling molecular pathogenesis from virus load (see 5C).

Indeed, analysis of post-mortem COVID-19 lung suggests two distinct stages of disease-progression (see Figure 5D). Early disease has high viral-load and high expression of cytokines and ISGs and sparse immune infiltrates, while in late disease, low viral loads, low local expression of cytokines and ISGs, and strong infiltration of macrophages and lymphocytes prevails. Patients who die early are unable to control SARS-CoV-2, while patients who die later suffer from diffuse tissue-damage and immunopathology (Nienhold et al., 2020) suggesting that late disease stage pathogenesis is apparently decoupled from acute viral-load.

##### 1.4.1.6 Multiple pathologies of COVID-19 phenotypes may converge mechanistically

The model demonstrates that excess activation of inflammatory signaling may turn productive inflammatory response and recruitment of immune cells into a detrimental cytokine storm and immunopathology. Importantly, such mechanisms can be triggered by an imbalance in *ACE2-DAK-B1R* signaling and associated regulatory components (e.g., miR200c or *SIRT1*) (see Figure 5 and Supplementary Table S1).

Altogether, the model suggests that dysregulation/disturbed homeostasis of eight mechanisms, alone or in combination, may contribute to the pathogenesis of major COVID-19 phenotypes. The multiple and seemingly unrelated clinical manifestations of COVID-19, including common disease symptoms (e.g., dry cough, myalgia, anosmia, transient diabetes and silent hypoxia) and severe manifestations (e.g., ARDS, lung fibrosis, acute coronary syndromes and thromboembolic events) may largely be linked to the pleiotropic activity of these core molecular players and mechanisms involved in the host response (see Figures 3, 4). Interestingly, the model reveals that some rarer phenotypes can be matched to other diseases sharing the same symptoms. Silent hypoxemia, for instance, causes the same symptoms as CCHS. The molecular pathologies of both converge on the same molecular mechanism (see Figures 3, 4).

##### 1.4.1.7 Validations, diagnostic and therapeutic implications

The rapid flow of new knowledge and information during the course of the pandemic enabled us to directly examine model-derived hypotheses in real-time, with several emerging as clinically validated or incorporated in specific multicenter studies. For example, COVID-19 organ damage often cannot be entirely explained by the virus’ organ tropism and local viral load. In COVID-19 associated kidney disease, for instance, viral load is low and unevenly distributed (Puelles et al., 2020; Su et al., 2020), and cannot explain the extensive kidney damage (Wang et al., 2021). These findings are in line with the proposed COVID-19 model. Currently, there are also no diagnostic tools that are associated with systemic pathophysiology of COVID-19. A systemic, virus independent disease mechanism requires systemic distribution of a signal that bears the potential to induce a broad spectrum of pathophysiological dysregulation in a variety of organs/tissues. A derailed pleiotropic signaling system such as the KKS/*B1R* signaling axis constitutes a likely candidate.

In this context, circulating MVs enriched in *B1R* and mir200c or circulating mir200c itself could serve as biomarker candidates. Indeed, serum, plasma or PBMC levels of miR200c has been identified as a diagnostic biomarker candidate in different COVID-19 related disease contexts, namely Kawasaki Disease (KWD) (Zhang et al., 2017), pneumonia (Liu et al., 2017), interstitial lung disease (Jiang et al., 2017), COPD (Cao et al., 2014) and fibrosis of multiple tissues (Yang et al., 2012; Ramachandran et al., 2013; Chen et al., 2017). It has also been shown that upregulated circulating miR-200c in plasma may increase the risk of obese individuals to severe COVID-19 (Papannarao et al., 2021).

In addition, the generated COVID-19 disease mechanism model contains target structures with implications for host-directed therapies. According to the model, pharmaceutically tractable target structures include the KKS at multiple levels such as *Kallikrein* inhibitors, *SerpinG1* enzyme replacement or *B1R* inhibitors for example, may represent a preferred therapeutic target, having been under evaluation for the treatment of hyperalgesia and osteoarthritis during the past decade. However, to date, no relevant clinical results have been published and most of the reported trials are inactive or have been stopped or suspended (see (Brock et al., 2022)). Interestingly, the induction of *B1R* also appears to be sensitive to treatment with dexamethasone. Modulation of *ACE2* activity represents another potential candidate for host directed therapy either by direct activation or indirectly by induction (e.g., through *SIRT-1* activators such as Melatonin, Resveratrol and Metformin or activators of *PPARγ* (Dambha-Miller et al., 2020)). Several of these are currently investigated and readouts of such trials utilizing drugs that potentially induce *ACE2* expression (Dambha-Miller et al., 2020) are summarized in the associated back-to-back paper (Brock et al., 2022).

## 3 Discussion

Our goal in creating the web-based *COVID-19 Explorer* was to provide an easily usable resource summarizing the key symptoms and molecular mechanisms associated with COVID-19 disease at the whole-patient level. To achieve this, we employed the Dataome technology in an iterative, expert-driven approach to:

• Build a comprehensive COVID-19 model
• Examine molecular mechanisms of specific individual symptoms
• Annotate relevant molecular components and pathways with supporting literature and observed evidence
• Visualize these findings, and share them with the community via a webserver that allows review of linked descriptive summaries
• Make hypotheses regarding the molecular etiology of both symptoms and disease and associated therapeutic strategies
• Monitor for the appearance of new findings that (in-) validate our original hypotheses, or modify accordingly

A key value of the COVID-19 Explorer is the way it permits the capture and contextualization of disease specific clinical and molecular information. With a plurality of emergent symptoms reported weekly during March-April 2020, it was critical to connect such clinical phenotypes and risk factors with both existing (e.g., from SARS-CoV-1) and emergent molecular findings. Our work highlights the speed at which bespoke clinico-molecular models can be built, emphasizing the important role that computer augmented disease modeling by domain experts can play in response to global health emergencies. Importantly, the COVID-19 model was built and organized into an updateable data framework driven by data capture, integration and curation activities. The model details molecular factors and systems that may drive COVID-19 and links the pleiotropic symptomology to possible underlying molecular pathology mechanisms. In comparison to other works, the ability to connect such disparate information layers facilitates a unique view in approaching COVID-19 at a whole patient, system-based level (Brock et al., 2022).

The COVID-19 Explorer is one of several valuable COVID-19 knowledge resources to emerge during the course of the pandemic. Prime examples of complementary open-source initiatives include COVID-19 UniprotKB (UniProt Consortium, 2021), Open Targets’ COVID-19 Target Prioritization Tool (Carvalho-Silva et al., 2018), and Reactome’s SARS-CoV-2 (COVID-19) infection pathway (Acencio, 2020). While these resources add significant value to our armamentarium of COVID-19 focused knowledgebases, the COVID-19 Explorer is the first to contextualize such data at the level of whole patient symptomatology. https://blog.opentargets.org/covid-19-target-prioritisation-tool-released/https://reactome.org/about/news/161-version-74-released.

Direct comparison of resources complicated by the diversity of starting motivations and utilities. For example, a recent large scale structural analysis has provided unique insights into complex and potentially important mechanisms regarding COVID-19, including viral protein self-assembly, molecular mimicry of human proteins, reversal of post-translational modifications, blockage of host translation, and the disabling of host defenses. In another instance, hospitalized COVID-19 patients were found to be positively correlated with (auto-) immune responses, not only providing confirmatory observations to our findings, but also highlighting the importance of laboratory-based validation of model-based hypotheses. By providing a whole patient perspective on the molecular etiology of COVID-19 symptomatology, our COVID-19 Explorer resource is complementary to the value.

Despite the broad utility of the whole patient model, several limitations exist. First, our integrated COVID-19 model was generated by a small team of biomedical experts, exploiting the content, technologies and processes of our proprietary Dataome solution. To do so, we partially relied on results gained from the extensive research that has been published on the original SARS coronavirus. For instance, the pathogen specific impact on the differential expression of ISG’s has been taken from work on SARS-CoV. We then used structured interaction and pathway data, as well as TDM and subsequent manual curation to identify upstream and downstream processes. All facts are supported by peer-reviewed literature and are transparently reported, though we cannot be certain about the accuracy or reproducibility of these results. We also screened for phenotypes that are associated with molecular perturbations (genetic or pharmacologic) of members within these regulatory networks. However, we limited this work to proposed key factors and the immediate regulatory elements.

Second, we also systematically collected COVID-19 associated phenotypes and risk profiles affecting different organ systems. Since a majority of these phenotypes also occur in other disease contexts, such as symptomatically related hereditary diseases, we identified the molecular mechanisms involved in their respective molecular etiologies. We then screened for convergence/divergence between the disturbed host mechanisms and those underlying the COVID-19 phenotypes. The advantage of such an approach is that key factors and interrelated pleiotropic regulatory concepts are quickly identified. However, our results likely need to be complemented by further systematic extension of the work. It seems logical, that the manifold symptoms and manifestations of COVID-19 result from the dysregulation of a few key elements converging in a pleiotropic mechanism which is connected to completely different phenotypes, depending on the individual tissue and organ context. While in many cases there is also multiple independent evidence linking the disturbed host system to the mechanisms underlying specific symptoms, the causal association with SARS-CoV-2 has yet to be proven. In that respect, we regard our approach as an effort to aggregate and interlink facts and connect existing knowledge that results in a defendable and testable hypothetical model that can inform future targeted research.

In terms of future directions, the current model is based on the molecular phenotype of the cells targeted and infected by SARS-CoV(-2) and provides a basis for explaining the diverse clinical phenotypes, observed risk factors and tractable strategies for therapeutic interventions and prevention. However, it does not provide a detailed mechanistic model for how direct pathogen host interactions induce and modulate the observed pathogen specific host response. As our initial approach was to reduce complexity by focusing on molecular phenotypes, we now have a consistent and testable base model that permits the systematic integration of additional data such as the full pathogen-host interactome. The model will also be further expanded by the +1-interaction level. The resulting network will then be further enriched by associated disease phenotypes from various sources, disease mechanisms and mode of actions of drugs targeting any of the components in the model. The resulting graph database will lend itself to the application of advance AI analytics to identify hypernodes and, eventually, mechanisms defining causality. This will provide novel angles to detect new strategies for intervention or for the comprehensive evaluation of existing interventional programs.

While our COVID-19 model was initially based on primarily data-mining hypotheses, data from more recent developments have helped update the initial model and also validate multiple predictions. For example, for some clinical phenotypes (e.g., KWD-like syndromes) predicted by our model it appeared that the clinical reality was ultimately superseding our model in real-time given the ongoing, massive global pandemic thereby providing timely validation (Brock et al., 2022). Moreover, our model revealed functionally connected mechanisms across various organ-systems, identified hypotheses for both, viral-dependent and -independent disease mechanisms, and associated pharmacologic targets that may warrant further evaluation (Brock et al., 2022). In this context our model, combined with other laboratory and/or real-world evidence, can be used both as a hypothesis generation and validation point regarding observed experimental or clinical findings.

In summary, our COVID-19 knowledge model links key molecular players in COVID-19 disease pathophysiology to common symptoms, severe manifestations and outcome/severity-associated risk factors at the whole patient level. We have validated that our COVID-19 Explorer provides a valuable and unique resource to support clinical and translational research audiences in hypothesis generation for new diagnostic and therapeutic strategies. We also anticipate that as the trajectory of scientific discovery continues to correlate the rate of technological advancement, biomedical research will grow increasingly dependent on similar human-focused and systems-based clinico-molecular information systems, capable of summarizing diverse findings in the form of intuitive whole patient disease models. Our work suggests that computer-augmented modelling of such knowledge by domain experts currently represents the most reliable approach in this regard. Moreover, it also provides a structured format through which future publication of expert reviews may be approached.

## Data Availability

Associated data are downloadable within dedicated sections of the web-based COVID-19 Explorer interface, accessible at http://covid19.molecularhealth.com. Additional data pertaining to this submission may also be made available upon reasonable request. In this context, it is important to emphasize that the development of the COVID-19 Explorer was enabled by MH’s Dataome platform, an expansive biomedical data and analytics infrastructure that contains a diverse and integrated array of open-source, proprietary and commercial data sources (over 100) and software (some licensed from third parties).

## References

[B1] Acencio M. L. (2020). SARS-CoV-2 Infection reactome. WHO. https://reactome.org/content/detail/person/0000-0002-8278-240X

[B2] Armaiz-Pena G. Allen J. K. Cruz A. Stone R. L. Nick A. M. Lin Y. G. (2013). Src activation by β-adrenoreceptors is a key switch for tumour metastasis. Nat. Commun. 4, 1403. 10.1038/ncomms2413 23360994 PMC3561638

[B3] Bohnert R. Vivas S. Jansen G. (2017). Comprehensive benchmarking of SNV callers for highly admixed tumor data. PLoS One 12, e0186175. 10.1371/journal.pone.0186175 29020110 PMC5636151

[B4] Brock S. Jackson D. B. Soldatos T. G. Hornischer K. Schäfer A. Diella F. (2022). Whole patient knowledge modeling of COVID-19 symptomatology reveals common molecular mechanisms. Front. Mol. Med. 2:1035290. 10.3389/fmmed.2022.1035290 PMC11285600 39086962

[B5] Brown E. G. (2004). Using MedDRA: Implications for risk management. Drug Saf. 27, 591–602. 10.2165/00002018-200427080-00010 15154830

[B6] Cao Z. Zha NgN. Lou T. Jin Y. Wu Y. Ye Z. (2014). microRNA-183 down-regulates the expression of BKCaβ1 protein that is related to the severity of chronic obstructive pulmonary disease. Hippokratia 18, 328–332. 26052199 PMC4453806

[B7] Carvalho-Silva D. O. Pierleoni A. Pignatelli M. Ong C. Fumis L. Karamanis N. (2018). Open targets platform: New developments and updates two years on. Nucleic Acids Res. 47, D1056–D1065. 10.1093/nar/gky1133 PMC6324073 30462303

[B8] Chen J. Cai J. Du C. Cao Q. Li M. Liu B. (2017). Recent advances in miR-200c and fibrosis in organs. Zhong Nan Da Xue Xue Bao Yi Xue Ban. 42, 226–232. 10.11817/j.issn.1672-7347.2017.02.018 28255128

[B9] Cherny N. I. Dafni U. Bogaerts J. Latino N. J. Pentheroudakis G. Douillard J. Y. (2017). ESMO-magnitude of clinical benefit scale version 1.1. Ann. Oncol. 28, 2340–2366. 10.1093/annonc/mdx310 28945867

[B10] Dambha-Miller H. Albasri A. Hodgson S. Wilcox C. R. Khan S. Islam N. (2020). Currently prescribed drugs in the UK that could upregulate or downregulate ACE2 in COVID-19 disease: A systematic review. BMJ Open 10, e040644. 10.1136/bmjopen-2020-040644 PMC7490921 32928868

[B11] Fernandez S. Lenoir C. Samer C. Rollason V. (2020). Drug interactions with apixaban: A systematic review of the literature and an analysis of VigiBase, the world health organization database of spontaneous safety reports. Pharmacol. Res. Perspect. 8, e00647. 10.1002/prp2.647 32881416 PMC7507549

[B12] Gonzalez N. Speir M. L. Schmelter D. Rosenbloom K. R. Raney B. J. (2021). The UCSC genome browser database: 2021 update. Nucleic Acids Res. 49, D1046–d1057. 10.1093/nar/gkaa1070 33221922 PMC7779060

[B13] Gordon D. E. Jang G. M. Bouhaddou M. Xu J. Obernier K. White K. M. (2020). A SARS-CoV-2 protein interaction map reveals targets for drug repurposing. Nature 583, 459–468. 10.1038/s41586-020-2286-9 32353859 PMC7431030

[B14] Harrison S. M. Biesecker L. G. Rehm H. L. (2019). Overview of specifications to the ACMG/AMP variant interpretation guidelines. Curr. Protoc. Hum. Genet. 103, e93. 10.1002/cphg.93 31479589 PMC6885382

[B15] Hirotsu Y. Schmidt-Edelkraut U. Nakagomi H. Sakamoto I. Hartenfeller M. Narang R. (2020). Consolidated BRCA1/2 variant interpretation by MH BRCA correlates with predicted PARP inhibitor efficacy association by MH guide. Int. J. Mol. Sci. 21, E3895. 10.3390/ijms21113895 PMC7312854 32486089

[B16] Howe K. L. E. Achuthan P. Allen J. Allen J. Armean I. M. Azov A. G. (2021). Ensembl 2021. Nucleic Acids Res. 49, D884–d891. 10.1093/nar/gkaa942 33137190 PMC7778975

[B17] Humphreys B. L. Del Fiol G. Xu H. (2020). The UMLS knowledge sources at 30: Indispensable to current research and applications in biomedical informatics. J. Am. Med. Inf. Assoc. 27, 1499–1501. 10.1093/jamia/ocaa208 PMC7647371 33059366

[B18] Jassal B. Matthews L. Viteri G. Gong C. Lorente P. Fabregat A. (2020). The reactome pathway knowledgebase. Nucleic Acids Res. 48, D498–d503. 10.1093/nar/gkz1031 31691815 PMC7145712

[B19] Jiang Z. Tao J. H. Zuo T. Li X. M. Wang G. S. Fang X. (2017). The correlation between miR-200c and the severity of interstitial lung disease associated with different connective tissue diseases. Scand. J. Rheumatol. 46, 122–129. 10.3109/03009742.2016.1167950 27309544

[B20] Kanehisa M. Furumichi M. Sato Y. Ishiguro-Watanabe M. Tanabe M. K. E. G. G. (2021). Kegg: Integrating viruses and cellular organisms. Nucleic Acids Res. 49, D545–d551. 10.1093/nar/gkaa970 33125081 PMC7779016

[B21] Kim S. P. Chen J. Cheng T. Gindulyte A. He J. He S. (2021). PubChem in 2021: New data content and improved web interfaces. Nucleic Acids Res. 49, D1388–d1395. 10.1093/nar/gkaa971 33151290 PMC7778930

[B22] Koh W. J. Anderson B. O. Carlson R. W. (2020). NCCN resource-stratified and harmonized guidelines: A paradigm for optimizing global cancer care. Cancer 126 (10), 2416–2423. 10.1002/cncr.32880 32348572

[B23] Krawczyk P. Święcicki Ł. (2020). ICD-11 vs. ICD-10 - a review of updates and novelties introduced in the latest version of the WHO International Classification of Diseases. Psychiatr. Pol. 54, 7–20. 10.12740/PP/103876 32447353

[B24] Kutmon M. van Iersel M. P. Bohler A. Kelder T. Nunes N. Pico A. R. (2015). PathVisio 3: An extendable pathway analysis toolbox. PLoS Comput. Biol. 11, e1004085. 10.1371/journal.pcbi.1004085 25706687 PMC4338111

[B25] Landrum M. J. Chitipiralla S. Brown G. R. Chen C. Gu B. Hart J. (2020). ClinVar: Improvements to accessing data. Nucleic Acids Res. 48, D835–D844. 10.1093/nar/gkz972 31777943 PMC6943040

[B26] Lipscomb C. E. (2000). Medical subject headings (MeSH)Medical subject headings (MeSH). Bull. Med. Libr. Assoc. 88, 265–266. 10928714 PMC35238

[B27] Liu Q. Du J. Yu X. Xu J. Huang F. Li X. (2017). miRNA-200c-3p is crucial in acute respiratory distress syndrome. Cell. Discov. 3, 17021. 10.1038/celldisc.2017.21 28690868 PMC5485385

[B28] Maglott D. Ostell J. Pruitt K. D. Tatusova T. (2011). Entrez gene: Gene-centered information at NCBI. Nucleic Acids Res. 39, D52–D57. 10.1093/nar/gkq1237 21115458 PMC3013746

[B29] Merabti T. Abdoune H. Letord C. Sakji S. Joubert M. Darmoni S. J. (2011). Mapping the ATC classification to the UMLS metathesaurus: Some pragmatic applications. Stud. Health Technol. Inf. 166, 206–213. 21685626

[B30] Nienhold R. Ciani Y. Koelzer V. H. Tzankov A. Haslbauer J. D. Menter T. (2020). Two distinct immunopathological profiles in autopsy lungs of COVID-19. Nat. Commun. 11, 5086. 10.1038/s41467-020-18854-2 33033248 PMC7546638

[B31] O'Leary N. Wright M. W. Brister J. R. Ciufo S. Haddad D. McVeigh R. (2016). Reference sequence (RefSeq) database at NCBI: Current status, taxonomic expansion, and functional annotation. Nucleic Acids Res. 44, D733–D745. 10.1093/nar/gkv1189 26553804 PMC4702849

[B32] Papannarao J. B. Schwenke D. O. Manning P. Katare R. (2021). Upregulated miR-200c may increase the risk of obese individuals to severe COVID-19. medRxiv.

[B33] Pradeep S. Huang J. Mora E. M. Nick A. M. Cho M. S. Wu S. Y. (2015). Erythropoietin stimulates tumor growth via EphB4. Cancer Cell. 28, 610–622. 10.1016/j.ccell.2015.09.008 26481148 PMC4643364

[B34] Puelles V. G. Lutgehetmann M. Lindenmeyer M. T. Sperhake J. P. Wong M. N. Allweiss L. (2020). Multiorgan and renal tropism of SARS-CoV-2. N. Engl. J. Med. 383, 590–592. 10.1056/NEJMc2011400 32402155 PMC7240771

[B35] Ramachandran S. Ilias Basha H. Sarma N. J. Lin Y. Crippin J. S. Chapman W. C. (2013). Hepatitis C virus induced miR200c down modulates FAP-1, a negative regulator of Src signaling and promotes hepatic fibrosis. PLoS One 8, e70744. 10.1371/journal.pone.0070744 23950995 PMC3741284

[B36] Sayers E. W. Bolton E. E. Brister J. R. Canese K. Chan J. Comeau D. C. (2021). Database resources of the national center for biotechnology information in 2023. Nucleic Acids Res. 49, gkac1032–d17. 10.1093/nar/gkac1032 PMC7778943 33095870

[B37] Schell M. J. Yang M. Teer J. K. Lo F. Y. Madan A. Coppola D. (2016). A multigene mutation classification of 468 colorectal cancers reveals a prognostic role for APC. Nat. Commun. 7, 11743. 10.1038/ncomms11743 27302369 PMC4912618

[B38] Schotland P. Racz R. Jackson D. B. Soldatos T. G. Levin R. Strauss D. G. (2021). Target adverse event profiles for predictive safety in the postmarket setting. Clin. Pharmacol. Ther. 109, 1232–1243. 10.1002/cpt.2074 33090463 PMC8246740

[B39] Soldatos T. G. Jackson D. B. (2019). Adverse event circumstances and the case of drug interactions. Healthc. (Basel) 7, E45. 10.3390/healthcare7010045 PMC6473808 30893930

[B40] Soldatos T. G. Taglang G. Jackson D. B. (2018). *In silico* profiling of clinical phenotypes for human targets using adverse event data. High. Throughput. 7, E37. 10.3390/ht7040037 PMC6306940 30477159

[B41] Su H. Yang M. Wan C. Yi L. X. Tang F. Zhu H. Y. (2020). Renal histopathological analysis of 26 postmortem findings of patients with COVID-19 in China. Kidney Int. 98, 219–227. 10.1016/j.kint.2020.04.003 32327202 PMC7194105

[B42] UniProt Consortium (2021). UniProt: The universal protein knowledgebase in 2021. Nucleic Acids Res. 49, D480–d489. 10.1093/nar/gkaa1100 33237286 PMC7778908

[B43] Ursu O. D. Holmes J. Bologa C. G. Yang J. J. Mathias S. L. Stathias V. (2018). DrugCentral 2018: An update. Nucleic Acids Res. 47, D963–d970. 10.1093/nar/gky963 PMC6323925 30371892

[B44] Varga Z. Flammer A. J. Steiger P. Haberecker M. Andermatt R. Zinkernagel A. S. (2020). Endothelial cell infection and endotheliitis in COVID-19. Lancet 395, 1417–1418. 10.1016/S0140-6736(20)30937-5 32325026 PMC7172722

[B45] Wang M. Xiong H. Chen H. Li Q. Ruan X. Z. (2021). Renal injury by SARS-CoV-2 infection: A systematic review. Kidney Dis. 7, 100–110. 10.1159/000512683 PMC7705946 33821207

[B46] Wishart D. S. Feunang Y. D. Guo A. C. Lo E. J. Marcu A. Grant J. R. (2018). DrugBank 5.0: A major update to the DrugBank database for 2018. Nucleic Acids Res. 46, D1074–d1082. 10.1093/nar/gkx1037 29126136 PMC5753335

[B47] Yang S. Banerjee S. de Freitas A. Sanders Y. Y. Ding Q. Matalon S. (2012). Participation of miR-200 in pulmonary fibrosis. Am. J. Pathol. 180, 484–493. 10.1016/j.ajpath.2011.10.005 22189082 PMC3349843

[B48] Yao X. Tsang T. Sun Q. Quinney S. Zhang P. (2020). Mining and visualizing high-order directional drug interaction effects using the FAERS database. BMC Med. Inf. Decis. Mak. 20, 50. 10.1186/s12911-020-1053-z PMC7079342 32183790

[B49] Zhang W. Wang Y. Zeng Y. Hu L. Zou G. (2017). Serum miR-200c and miR-371-5p as the useful diagnostic biomarkers and therapeutic targets in Kawasaki disease. Biomed. Res. Int. 2017, 8257862. 10.1155/2017/8257862 28656149 PMC5471556

