# Supplementary material for: The COVID-19 explorer—An integrated, whole patient knowledge model of COVID-19 disease
Source: Front Mol Med. 2022 Dec 22;2:1035215. doi: 10.3389/fmmed.2022.1035215 (PMC11285624; doi:10.3389/fmmed.2022.1035215)
Supplement: Supplementary file 1 [file DataSheet2.pdf]

Quick Guide

MH Corona Explorer

Release 3.0

Quick Guide Rev. 1

12/18/2020

Contents

1. ABOUT MH CORONA EXPLORER 3
2. GETTING STARTED 4
Logging in 4
Terms and concepts used 4
3. ORGANS & SYMPTOMS: THE 3D HUMAN MODEL 5
Navigating with a mouse, trackpad, or touchpad 6
Navigating with the computer keyboard 6
Navigating with the built-in toolbars 7
4. PATHOGENIC MECHANISMS TAB 8
5. DISEASE MODEL TAB 9
6. EXPANDED MODEL TAB 10
7. SYMPTOM MECHANISMS TAB 11
Detailed Symptom Mechanism 12
8. FUNCTIONAL MECHANISMS TAB 13
Detailed Functional Mechanism 13
9. PUBLICATIONS TAB 14
10. TERMINOLOGY REFERENCE TAB 14
11. PROJECT BACKGROUND AND FURTHER INFORMATION 15
Contact Molecular Health 15

Molecular Health GmbH
Kurfuersten-Anlage 21,
69115 Heidelberg, Germany
T +49 6221 43851-0
germany@molecularhealth.com
Better data. Better insights.
Better outcomes.
molecularhealth.com
CORONA EXPLORER

1. About MH Corona Explorer

MH Corona Explorer is an interactive bioinformatics software application for analyzing the effects of the SARS-CoV-2 virus, from the symptom level down to the protein/gene level.

You can use it to understand the diseases and symptoms triggered by the virus, and to understand potential treatments (drugs) and vaccines, by looking at how and where they interact in the cellular mechanisms.

The core data in the MH Corona Explorer comes from analysis of over 10,000 relevant publications by MH bioinformaticians, curators, and medical experts.

MH Corona Explorer visualizes the mechanisms underlying the SARS-CoV-2 virus in eight interactive screens:

- **Organs & Symptoms**: explore a 3D human model for an overview of organs, diseases, and symptoms, and follow links to the underlying biomedical models.
- **Pathogenic Mechanisms**: explore an interactive network of organs, symptoms, mechanisms, and signaling axes.
- **Disease Model**: understand central mechanisms behind cell damage, even in cells not directly infected by the virus.
- **Expanded Model**: visualize pathways, molecular mechanisms, and biological systems affected by the virus.
- **Symptom Mechanisms**: interactive list of symptom mechanisms and the related genes and proteins.
- **Functional Mechanisms**: list of mechanisms triggered by the SARS-CoV-2 virus, and the related genes and proteins.
- **Publications**: Browse over 300 publications underlying the MH Corona Explorer.
- **Terminology Reference**: a resource that links terminology from the disparate disciplines of biology and medicine.

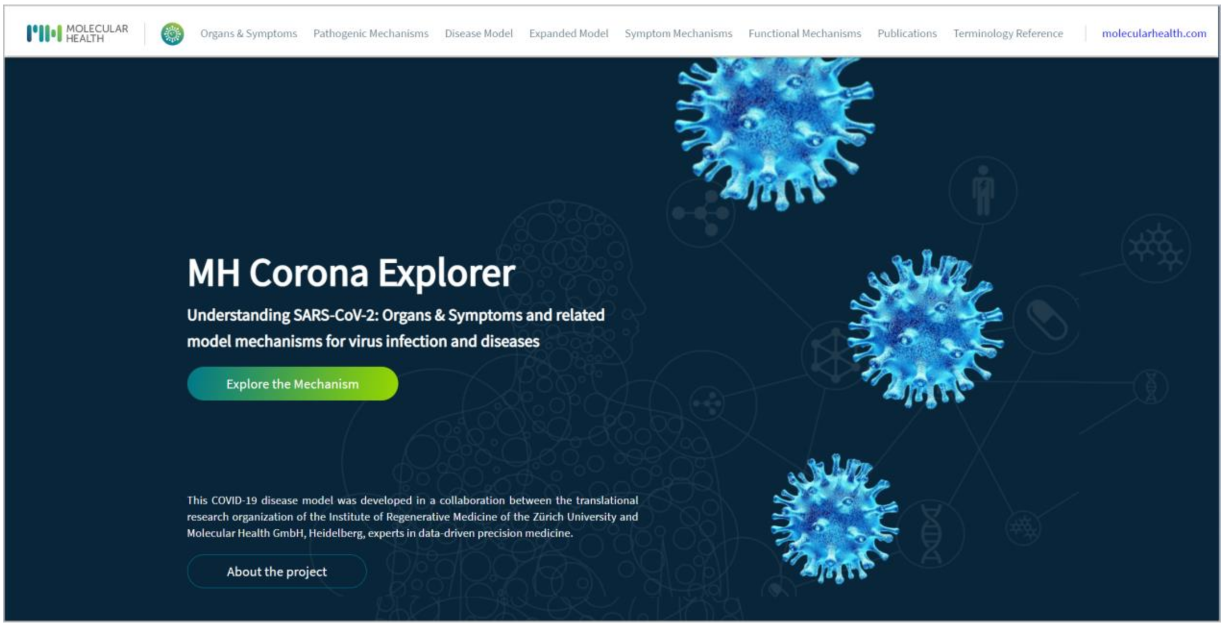

Molecular Health GmbH
Kurfuersten-Anlage 21,
69115 Heidelberg, Germany
T +49 6221 43851-0
germany@molecularhealth.com

2. Getting started

Logging in

Login: use the URL https://covid19.molecularhealth.com and enter your login credentials.

Terms and concepts used

Depending on whether you access the MH Corona Explorer as a physician, as a biologist, or as a user not trained as a scientific expert, you may be used to a certain specialized terminology. The screens in MH Corona Explorer use medical terms or terms from the domain of biology, or both, as required by the context. You can use the **Terminology Reference** section to understand the relationship between terms.

Disease descriptions often use a medical, academic term sometimes referring to the organ (Example: "*Cardio Vascular Manifestation*" for the *Cardio Vascular System)* or disturbance of the organ (e.g. *Ageusia*)

Symptoms and syndromes: For the sake of simplicity, to reduce the number of terms used, we use the term "symptom" for individual symptoms (e.g. *thrombosis*) as well as for groups of symptoms often referred to as a syndrome. We use "disease" to define a group of symptoms with a specific cause.

Mechanisms focus on key biological mechanisms (e.g. *coagulation*), for this reason they do not share the same name as the symptoms they cause. For example, while "*pneumonitis*" is the term used to describe a symptom of lung inflammation, the term used for this mechanism is "inflammatory signaling".

Gene names and molecule names: In some cases, different names are used in parallel to denote the same genes. Here are some common examples: the gene name DABK is a synonym for the molecule [des-Arg9] BK, the gene name BDKRB1 is a synonym for B1R, often used in biological models, and KNG1 is a synonym of HK. Where feasible, to facilitate understanding, we list both versions.

? Help texts and legends: Click the question mark symbol (?) at the top right of each screen to access an explanation of the screen as well as an explanatory list of any symbols used.

Description: Click **Mechanism Description** or **Model Description** to see a detailed explanation of the model or the mechanism.

Quick Guide: Click **Explorer Quick Guide** on any screen to download this Quick Guide as a PDF:

Molecular Health GmbH
Kurfuersten-Anlage 21,
69115 Heidelberg, Germany
T +49 6221 43851-0
germany@molecularhealth.com
Better data. Better insights.
Better outcomes.
molecularhealth.com
CORONA EXPLORER

3. Organs & Symptoms: The 3D Human Model

The 3D human model on the **Organs & Symptoms** tab is a highly interactive model for illustrating how the SARS-CoV-2 infection affects each organ of the human body. You can rotate and move the model, zoom in and out, see behind anatomical structures, add labels, and write or draw on the model.

See Navigating with a mouse, trackpad, or touchpad for details on exploring the human 3D model.

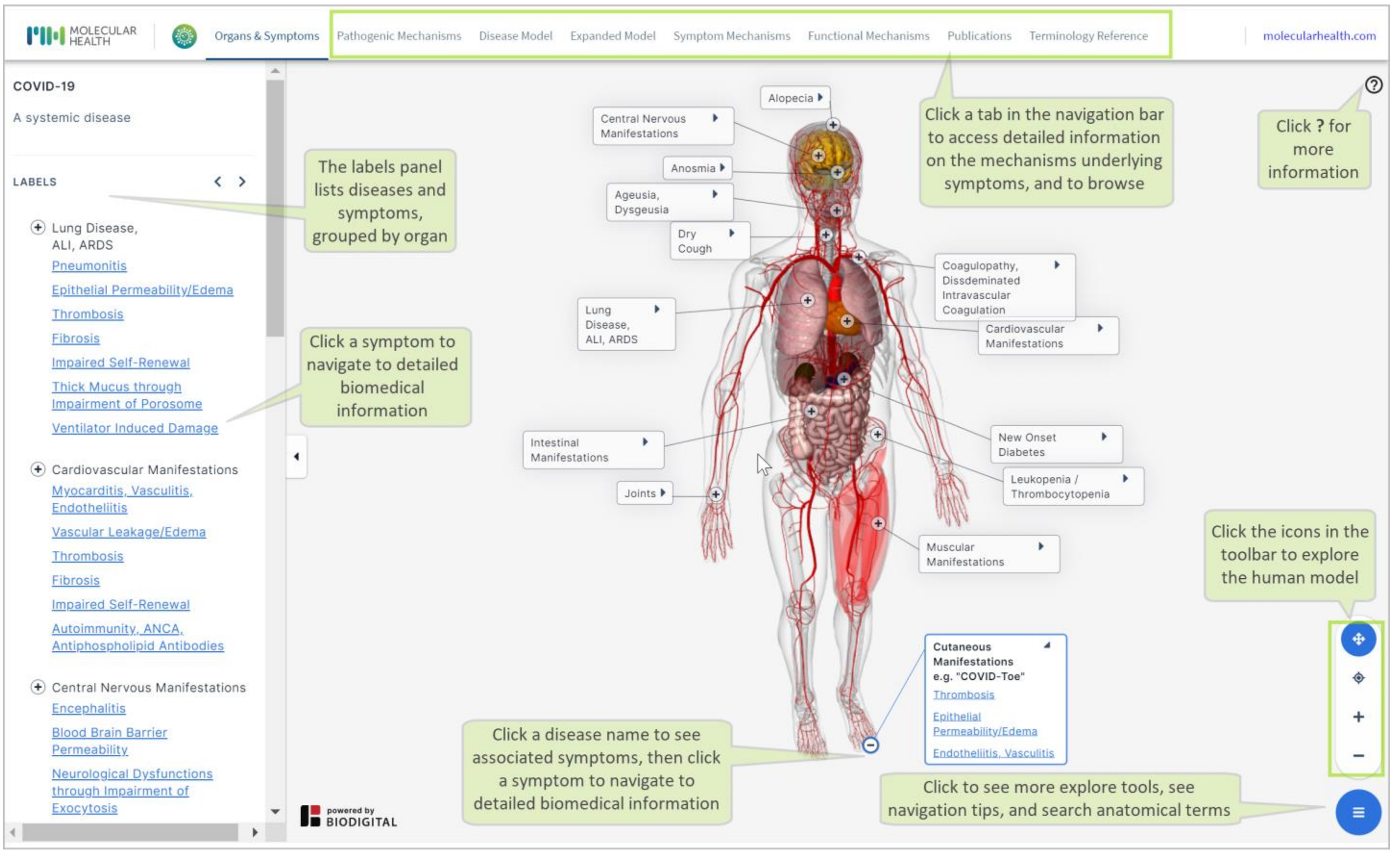

Molecular Health GmbH
Kurfuersten-Anlage 21,
69115 Heidelberg, Germany
T +49 6221 43851-0
germany@molecularhealth.com

Navigating with a mouse, trackpad, or touchpad

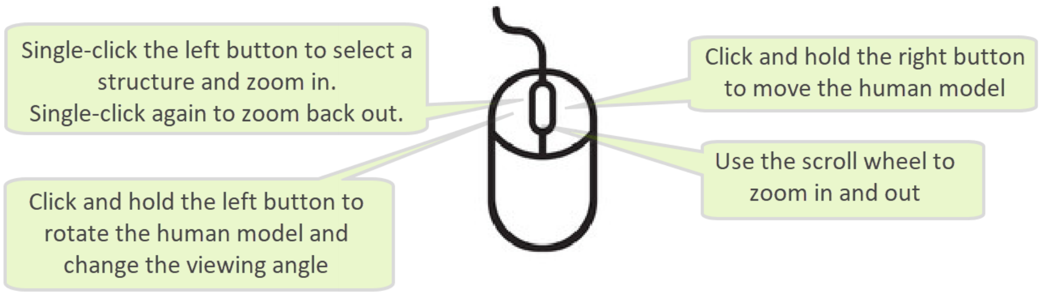

With a touchpad, you can also swipe with two fingers to zoom in and out.

Navigating with the computer keyboard

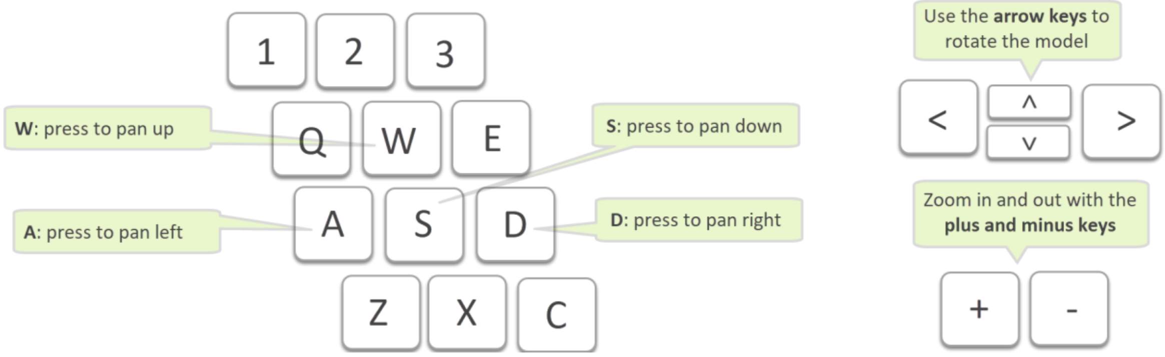

To move in smaller steps, hold the shift key while using the above controls.

Molecular Health GmbH
Kurfuersten-Anlage 21,
69115 Heidelberg, Germany
T +49 6221 43851-0
germany@molecularhealth.com

Better data. Better insights.
Better outcomes.
molecularhealth.com
CORONA EXPLORER

Navigating with the built-in toolbars

Here is an overview of the available tools. As the toolbars are task-sensitive, the entire selection of tools is not visible on the screen at any one time.

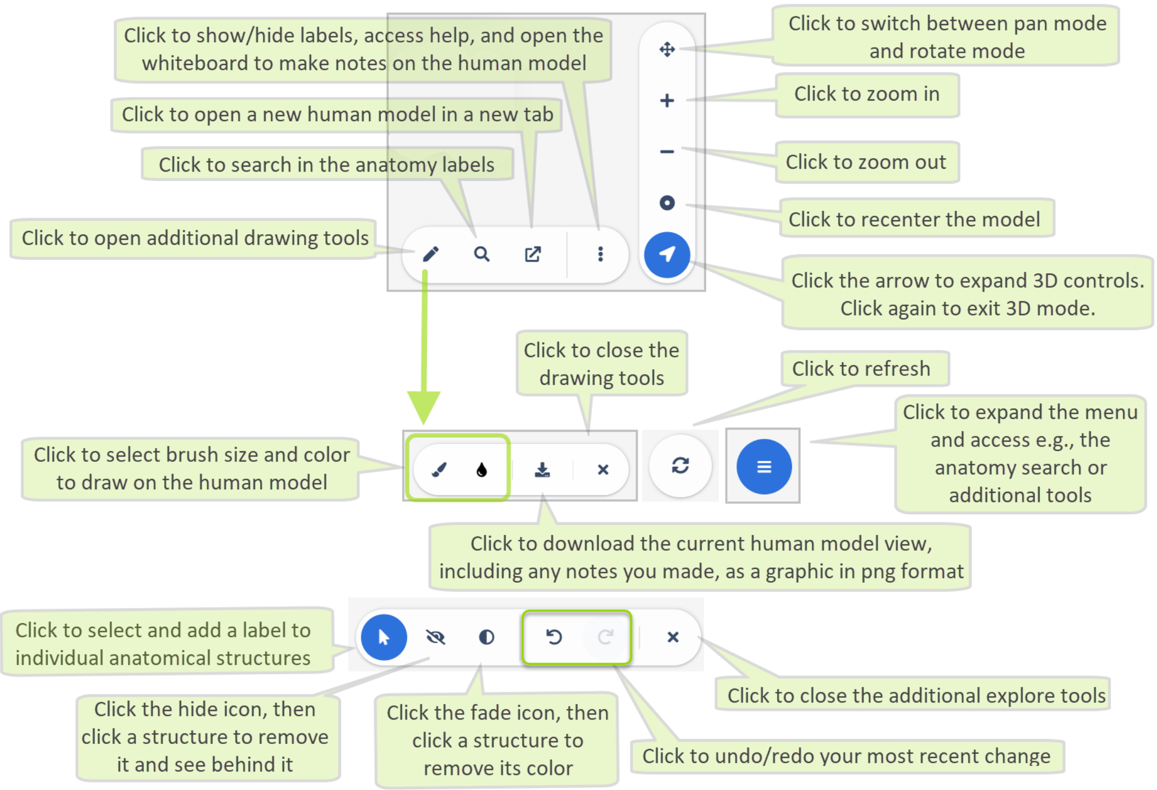

Molecular Health GmbH
Kurfuersten-Anlage 21,
69115 Heidelberg, Germany
T +49 6221 43851-0
germany@molecularhealth.com
Better data. Better insights.
Better outcomes.
molecularhealth.com
CORONA EXPLORER

4. Pathogenic Mechanisms tab

On the **Pathogenic Mechanisms** tab, you can explore eight interactive networks showing different perspectives on the mechanisms involved in an infection with the SARS-CoV-2 virus. Scroll through the screens to see organ-specific disease manifestations, pathogenic mechanisms, and associated symptoms. You can visualize how individual entities interact along signaling axes, and navigate to detailed information on each mechanism.

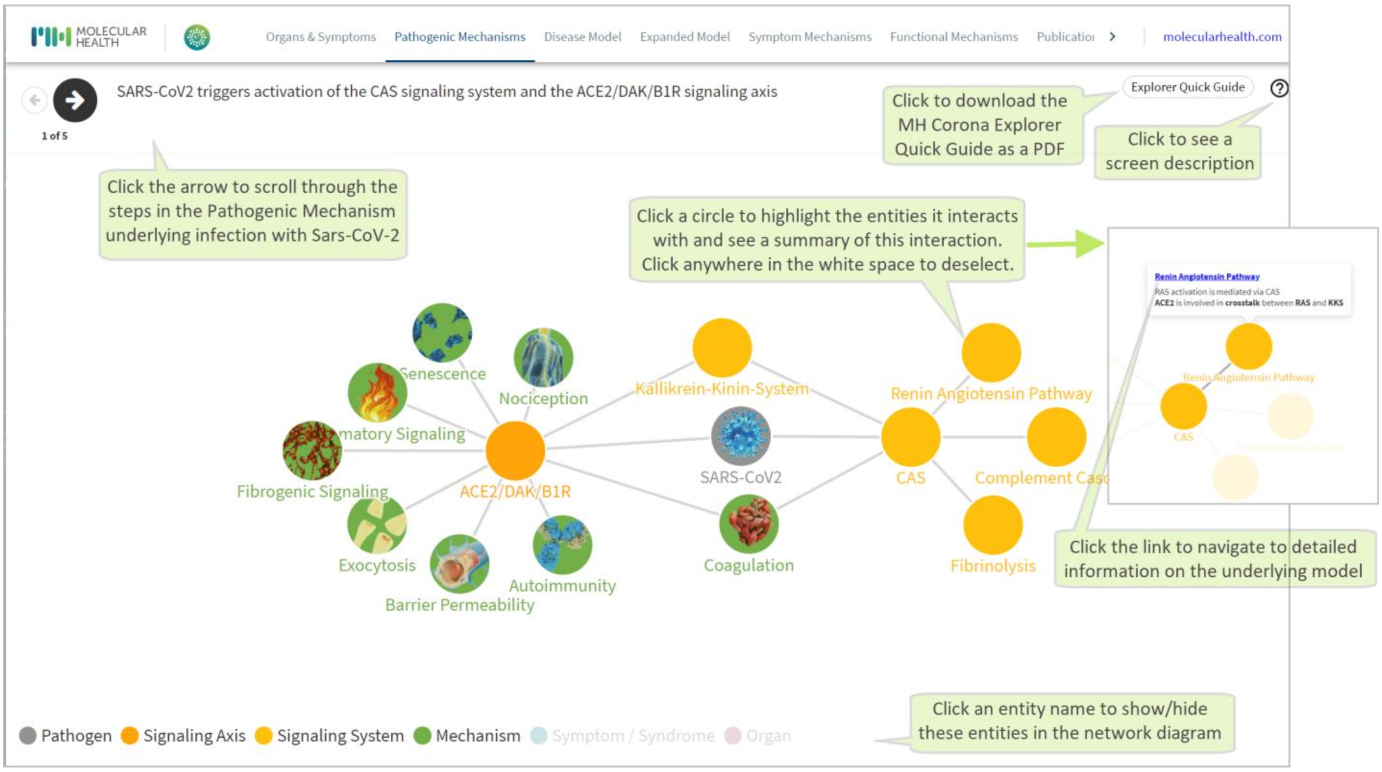

To avoid overcrowding the network diagram, ″inflammation″ is not shown.

Molecular Health GmbH
Kurfuersten-Anlage 21,
69115 Heidelberg, Germany
T +49 6221 43851-0
germany@molecularhealth.com

Better data. Better insights.
Better outcomes.
molecularhealth.com

5. Disease Model tab

The **Disease Model** tab, **Cell mechanism** screen shows key mechanisms of the virus that cause damage at the cell level.

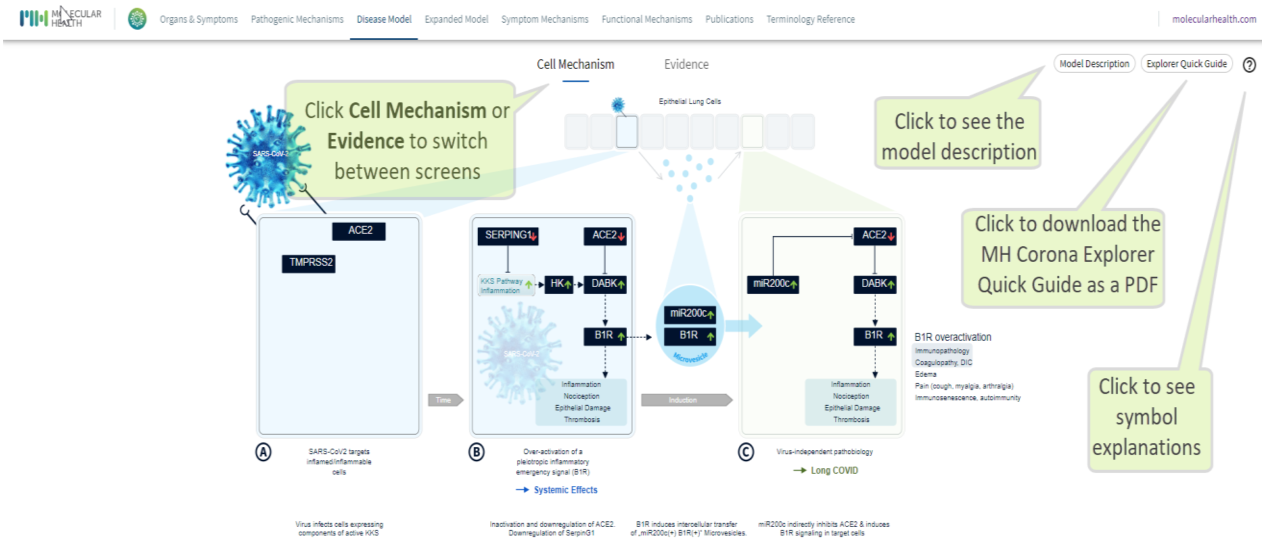

The **Disease model** tab, **Evidence** screen shows short and long term consequences observed in a group of SARS-CoV-2 patients. The Mechanisms are described in detail pressing the Button « Show Description »

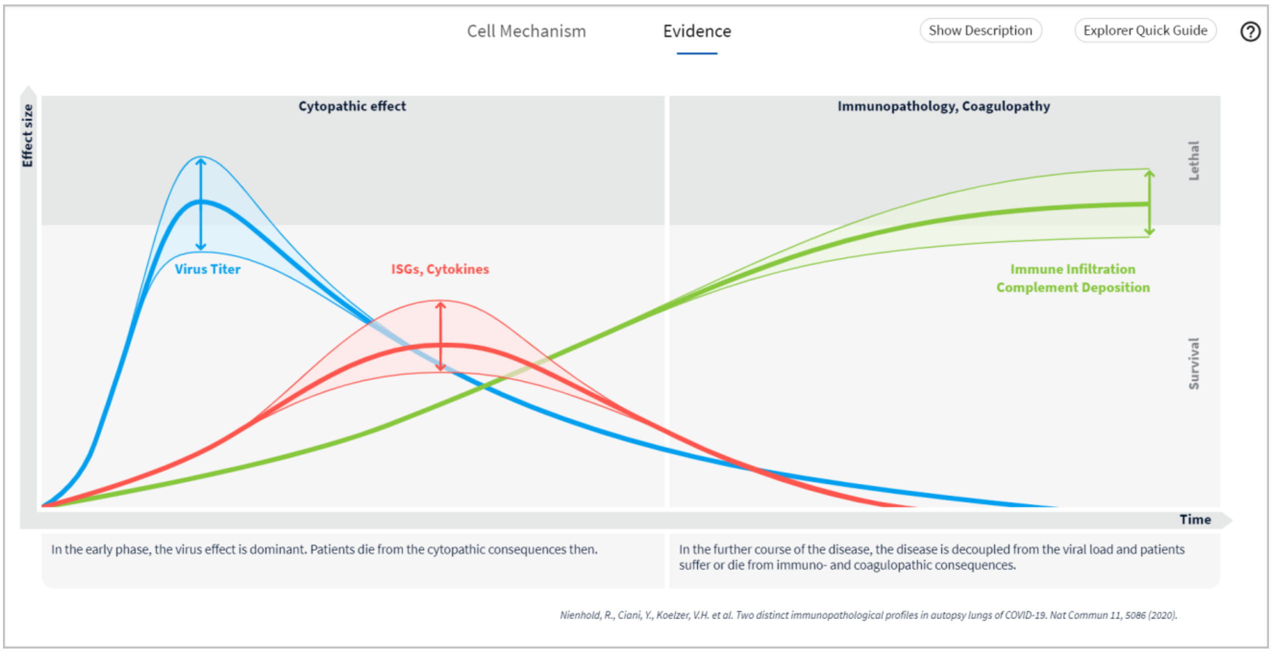

6. Expanded Model tab

The **Expanded Model** tab shows the systems and pathways involved in SARS-CoV-2, and provides links to detailed visualizations of the pathways involved in the underlying **Symptom Mechanisms** and **Functional Mechanisms**.

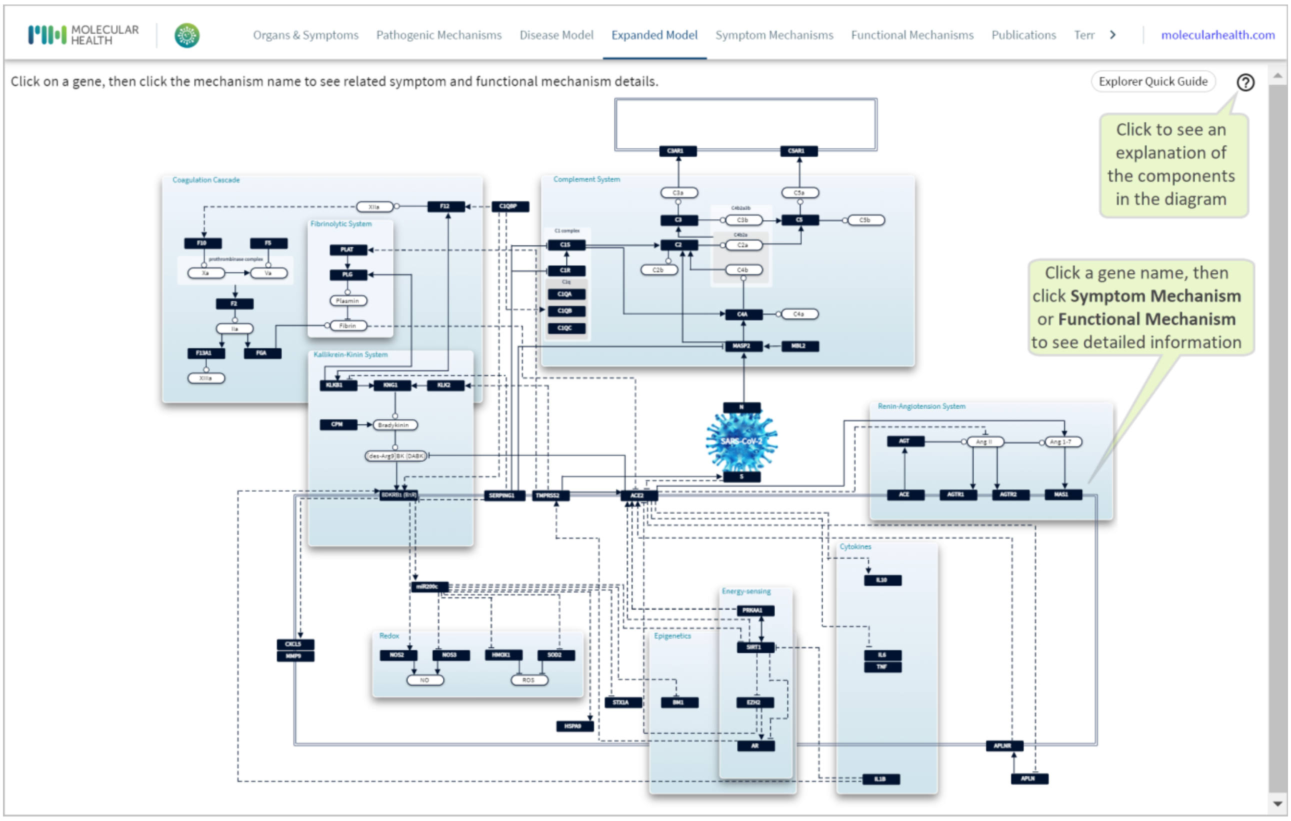

Molecular Health GmbH
Kurfuersten-Anlage 21,
69115 Heidelberg, Germany
T +49 6221 43851-0
germany@molecularhealth.com

7. Symptom Mechanisms tab

The Symptom Mechanisms tab shows the mechanisms involved in SARS-CoV-2 and provides links to associated pathways. This information is curated by MH and includes links to underlying publications.

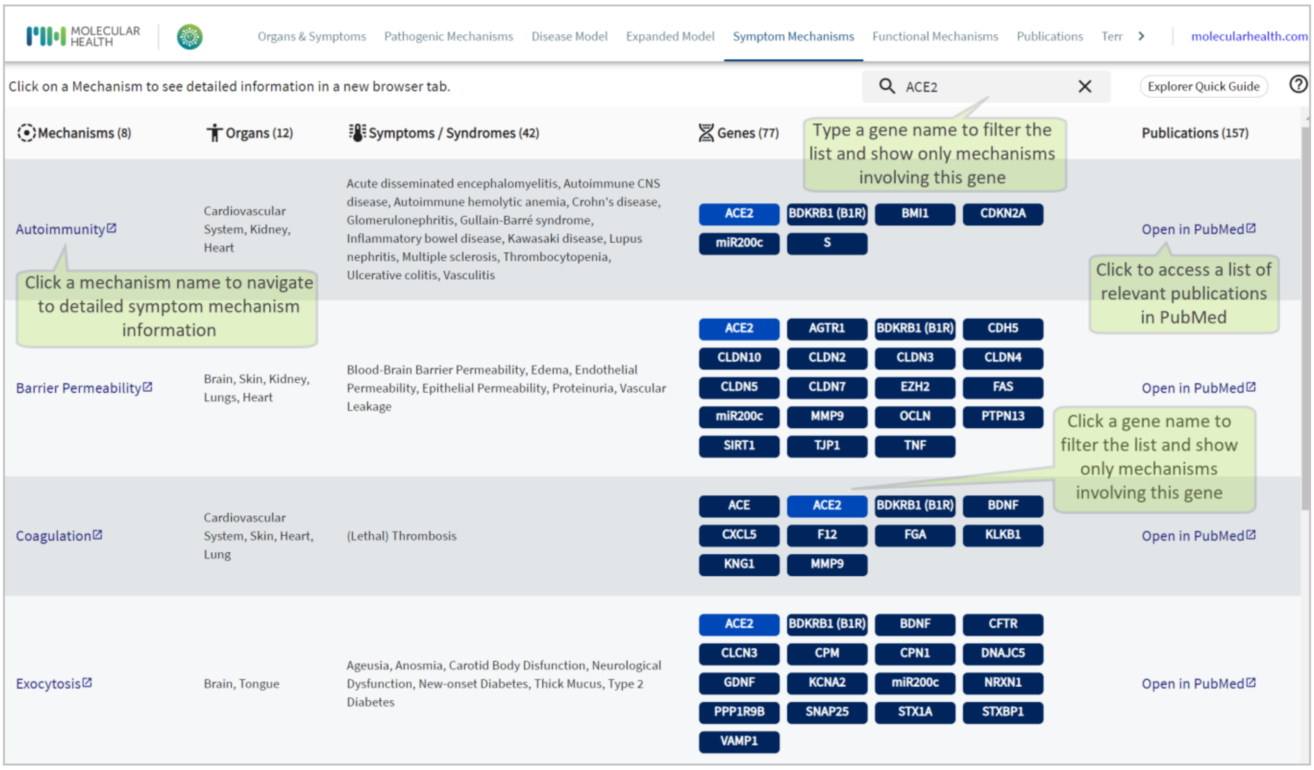

Molecular Health GmbH
Kurfuersten-Anlage 21,
69115 Heidelberg, Germany
T +49 6221 43851-0
germany@molecularhealth.com

Better data. Better insights.
Better outcomes.
molecularhealth.com

CORONA EXPLORER

Detailed Symptom Mechanism

Here is an example of a detailed **Symptom Mechanism**.

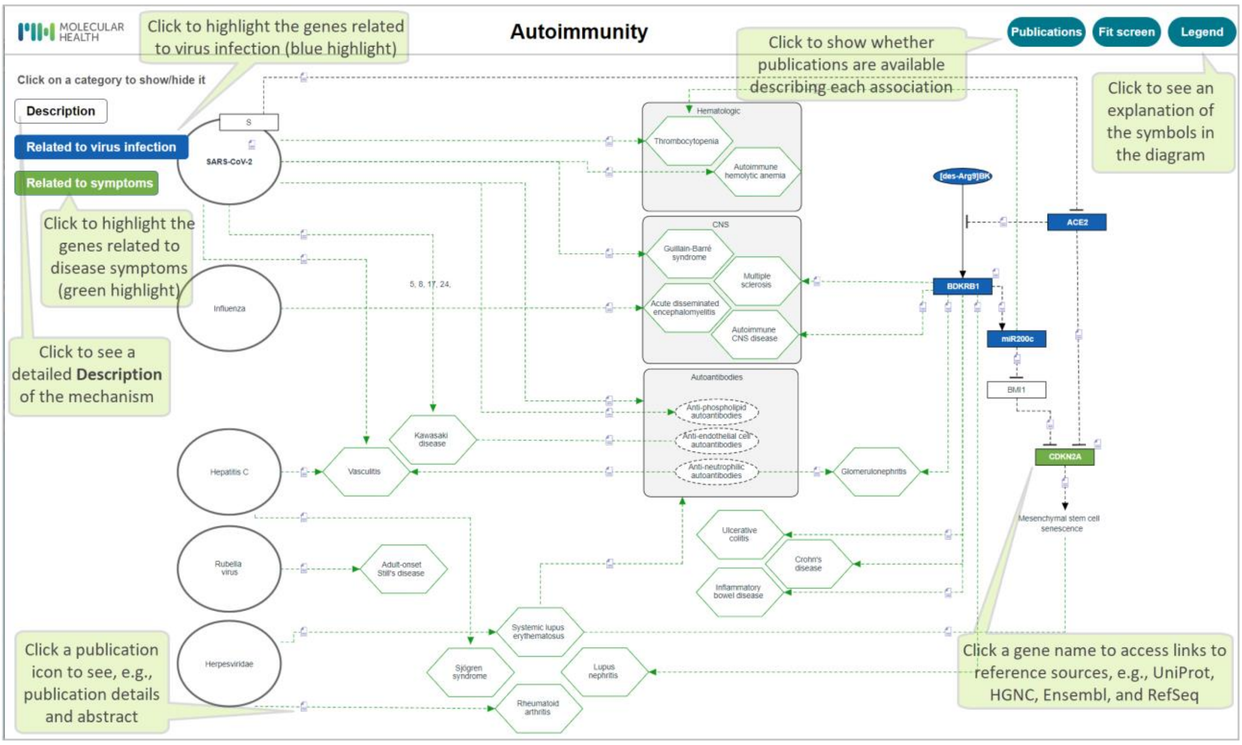

Molecular Health GmbH
Kurfuersten-Anlage 21,
69115 Heidelberg, Germany
T +49 6221 43851-0
germany@molecularhealth.com

Better data. Better insights.
Better outcomes.
molecularhealth.com

CORONA EXPLORER

8. Functional Mechanisms tab

The **Functional Mechanisms** tab lists the mechanisms involved in SARS-CoV-2 and provides links to associated pathways. This information is curated by MH and includes links to underlying publications.

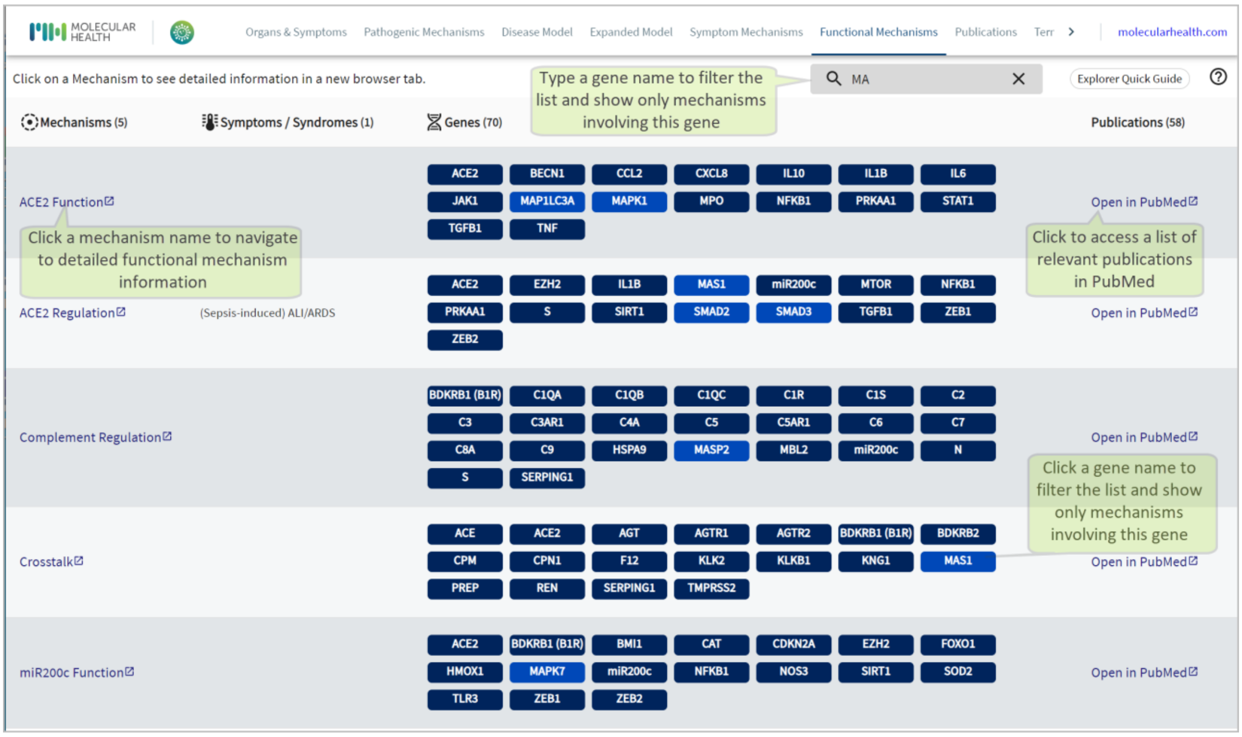

Detailed Functional Mechanism

Here is an example of a detailed **Functional Mechanism**.

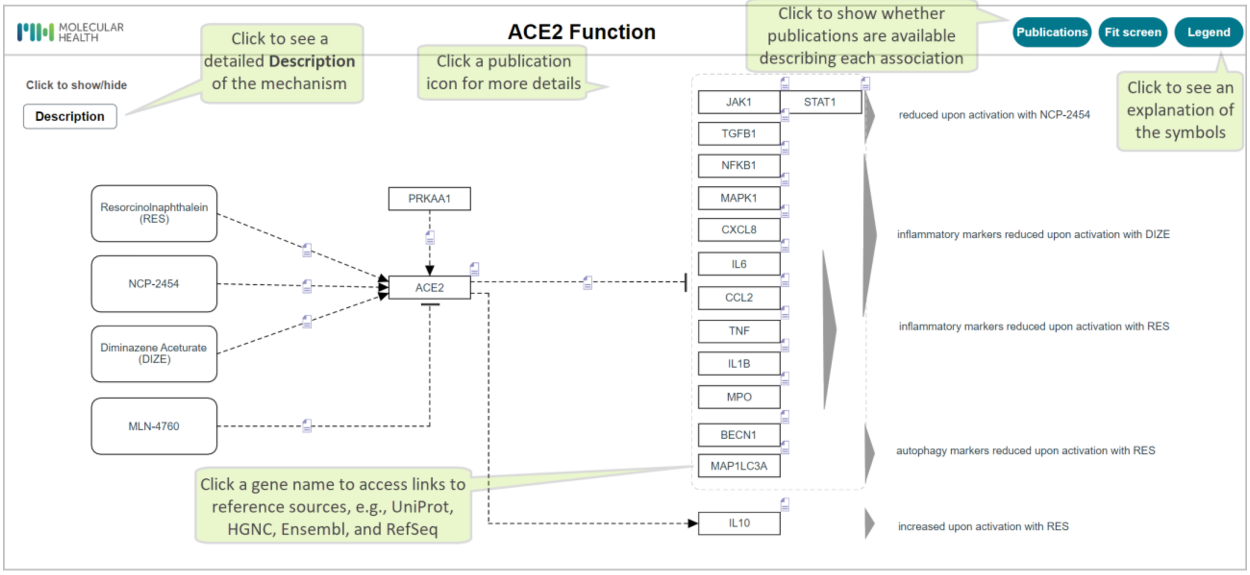

9. Publications tab

The **Publications** tab provides a list of relevant publications curated by MH experts and used to create all models and mechanisms. The default sort order of the **Publications** list is by PMID, with the most recent publication (the largest PMID number) first.

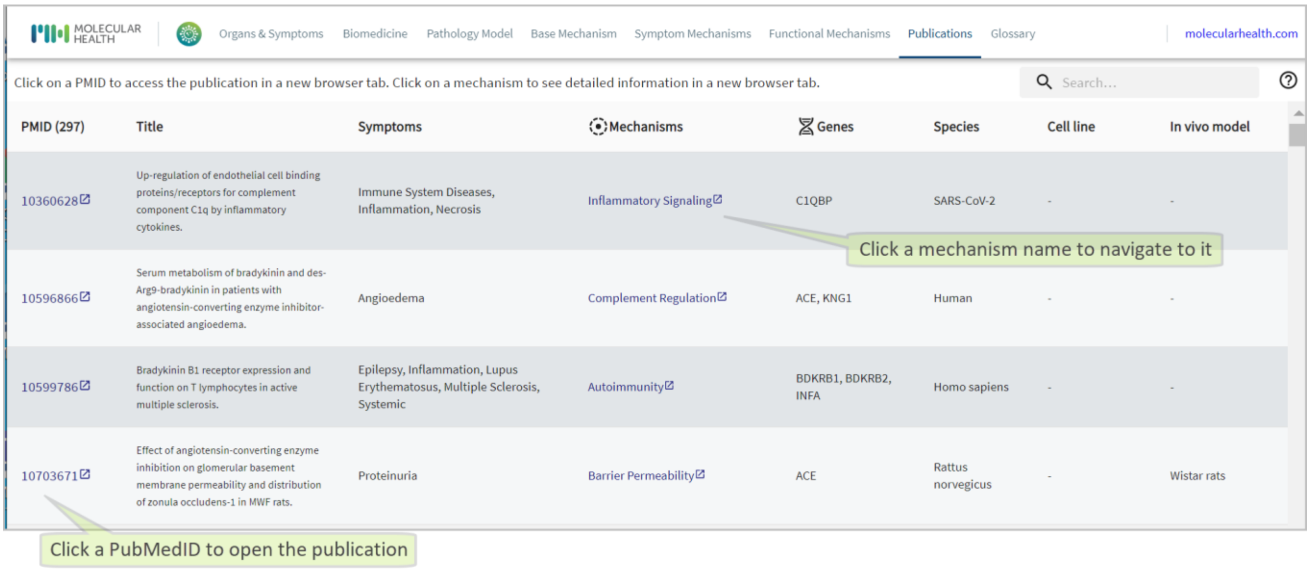

PMID (297)	Title	Symptoms	Mechanisms	Genes	Species	Cell line	In vivo model
10360628	Up-regulation of endothelial cell binding proteins/receptors for complement component C1q by inflammatory cytokines.	Immune System Diseases, Inflammation, Necrosis	Inflammatory Signaling	C1QBP	SARS-CoV-2	-	-
10596866	Serum metabolism of bradykinin and des-Arg9-bradykinin in patients with angiotensin-converting enzyme inhibitor-associated angioedema.	Angioedema	Complement Regulation	ACE, KNG1	Human	-	-
10599786	Bradykinin B1 receptor expression and function on T lymphocytes in active multiple sclerosis.	Epilepsy, Inflammation, Lupus Erythematosus, Multiple Sclerosis, Systemic	Autoimmunity	BDKRB1, BDKRB2, INFA	Homo sapiens	-	-
10703671	Effect of angiotensin-converting enzyme inhibition on glomerular basement membrane permeability and distribution of zonula occludens-1 in MWF rats.	Proteinuria	Barrier Permeability	ACE	Rattus norvegicus	-	Wistar rats

10. Terminology Reference tab

Find associated terms from the domains of biology and medicine using the search function. The terms are grouped by organ and sorted from head to toe.

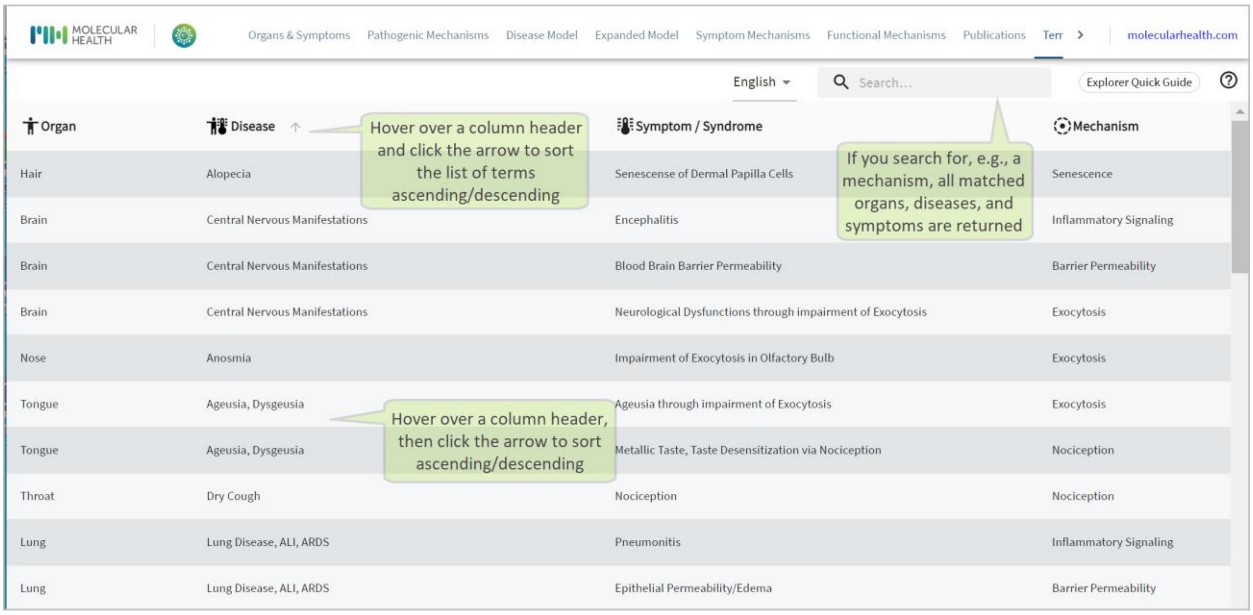

Organ	Disease	Symptom / Syndrome	Mechanism
Hair	Alopecia	Senescense of Dermal Papilla Cells	Senescence
Brain	Central Nervous Manifestations	Encephalitis	Inflammatory Signaling
Brain	Central Nervous Manifestations	Blood Brain Barrier Permeability	Barrier Permeability
Brain	Central Nervous Manifestations	Neurological Dysfunctions through impairment of Exocytosis	Exocytosis
Nose	Anosmia	Impairment of Exocytosis in Olfactory Bulb	Exocytosis
Tongue	Ageusia, Dysgeusia	Ageusia through impairment of Exocytosis	Exocytosis
Tongue	Ageusia, Dysgeusia	Metallic Taste, Taste Desensitization via Nociception	Nociception
Throat	Dry Cough	Nociception	Nociception
Lung	Lung Disease, ALI, ARDS	Pneumonitis	Inflammatory Signaling
Lung	Lung Disease, ALI, ARDS	Epithelial Permeability/Edema	Barrier Permeability

Molecular Health GmbH
Kurfuersten-Anlage 21,
69115 Heidelberg, Germany
T +49 6221 43851-0
germany@molecularhealth.com

Better data. Better insights.
Better outcomes.
molecularhealth.com

CORONA EXPLORER

11. Project background and further information

This COVID-19 disease model was developed as a collaboration between the translational research organization of the Institute of Regenerative Medicine of the Zürich University and Molecular Health GmbH, Heidelberg, experts in data-driven precision medicine.

The disease model is based on the Augmented Clinico-Molecular Disease Modeling technology (aCMDM), linking key molecular entities to COVID-19 pathophysiology and to disease symptoms (e.g., dry cough, myalgia, anosmia, dysgeusia/ageusia, new onset diabetes, etc.), severe manifestations (e.g., ARDS, ALI, lung fibrosis, cardiovascular complications), and outcome- and severity-associated risk factors (e.g., age, sex, smoking, comorbidities).

Where can I find more information?

For further details see “Unraveling the Multiple Manifestations of COVID-19 using Augmented Clinico-Molecular Disease Modeling” (submitted for publication) or contact us at corona@molecularhealth.com

Contact Molecular Health

You can contact the Molecular Health Customer Service team by email at corona@molecularhealth.com.

Molecular Health GmbH
Kurfuersten-Anlage 21,
69115 Heidelberg, Germany
T +49 6221 43851-0
germany@molecularhealth.com
