# Supplementary figures and images for: The COVID-19 explorer—An integrated, whole patient knowledge model of COVID-19 disease
Source: Front Mol Med. 2022 Dec 22;2:1035215. doi: 10.3389/fmmed.2022.1035215 (PMC11285624; doi:10.3389/fmmed.2022.1035215)

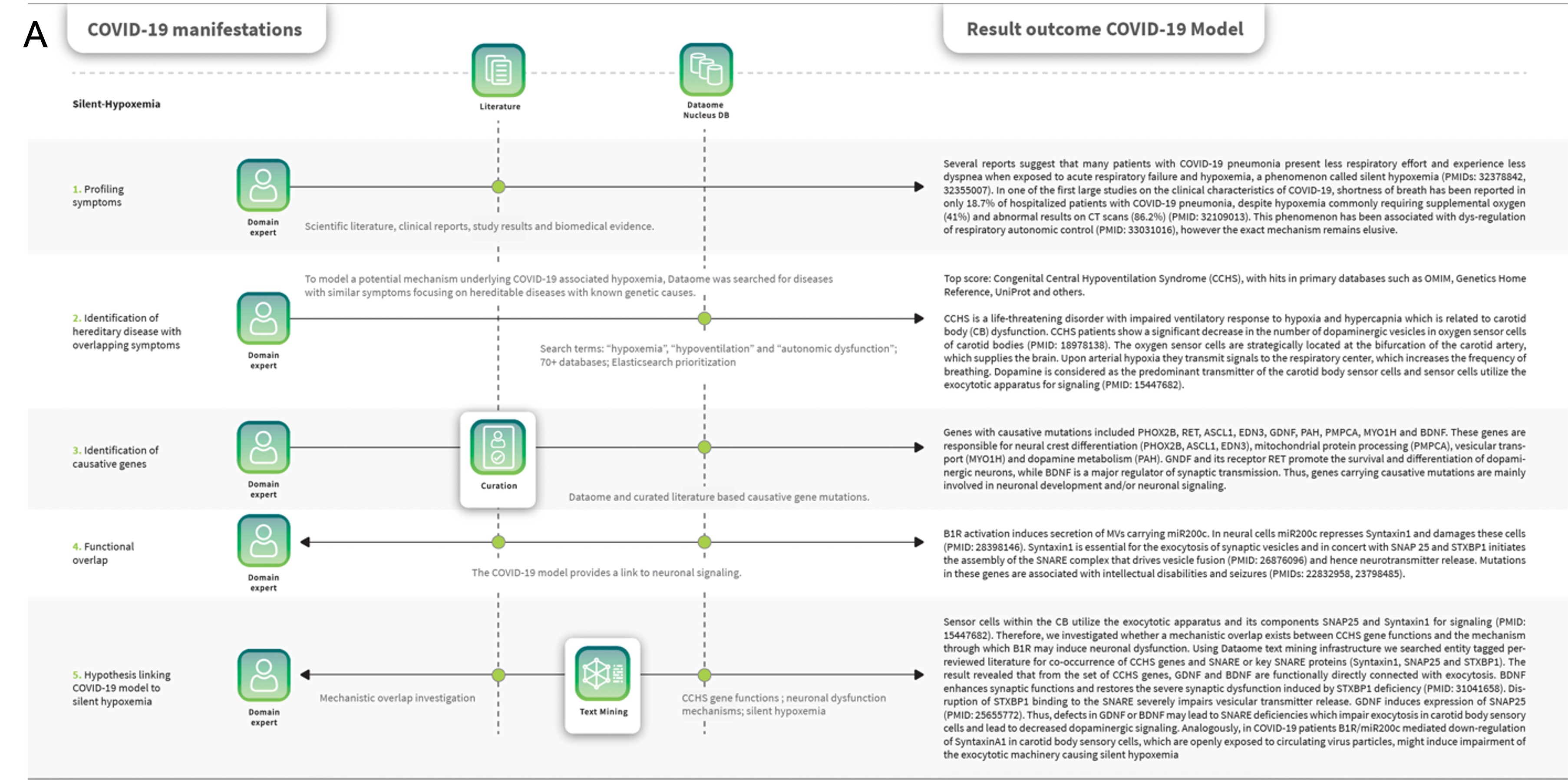

Supplement: Supplementary file 2 [file Image3.jpg]

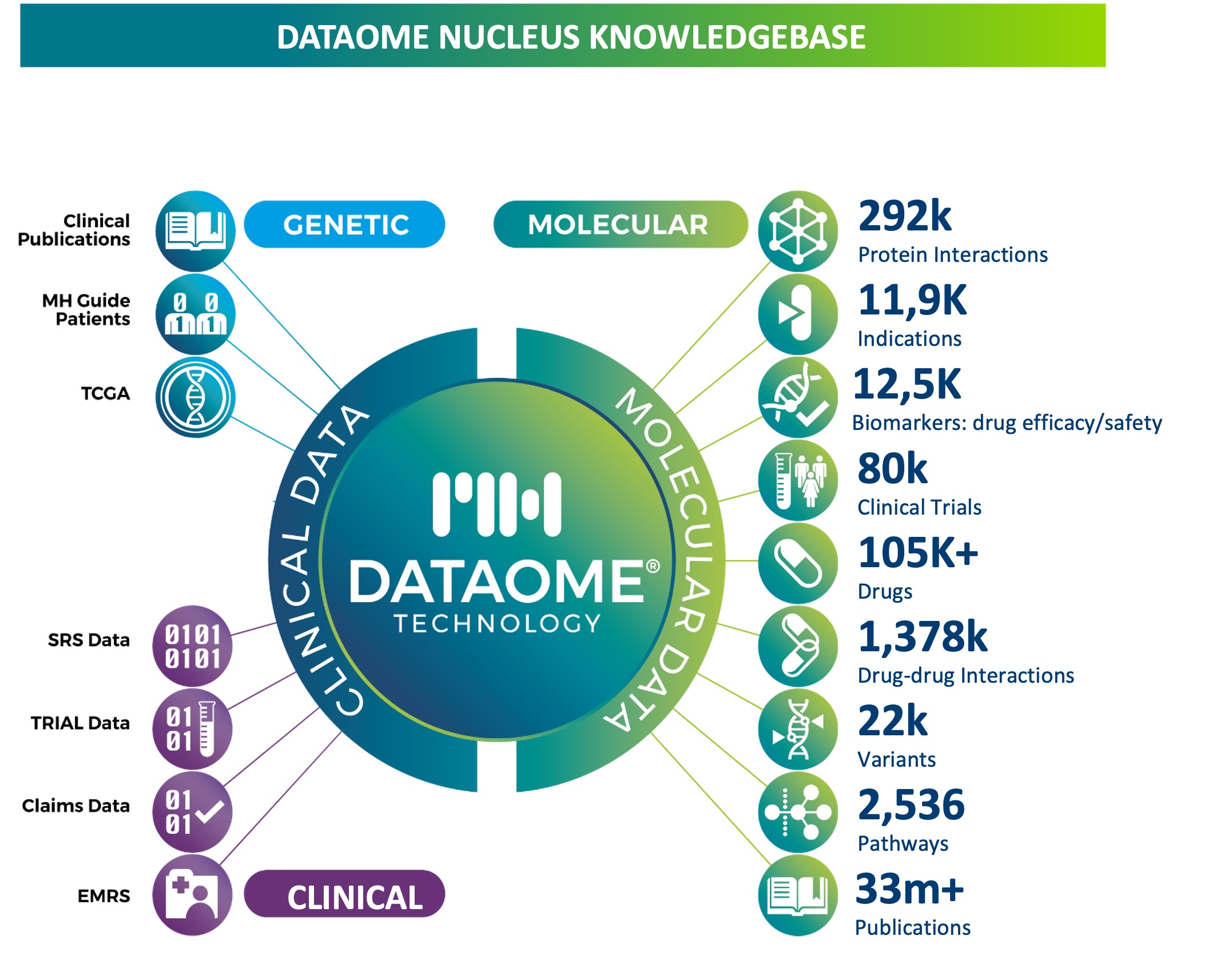

Supplement: Supplementary file 3 [file Image2.jpg]

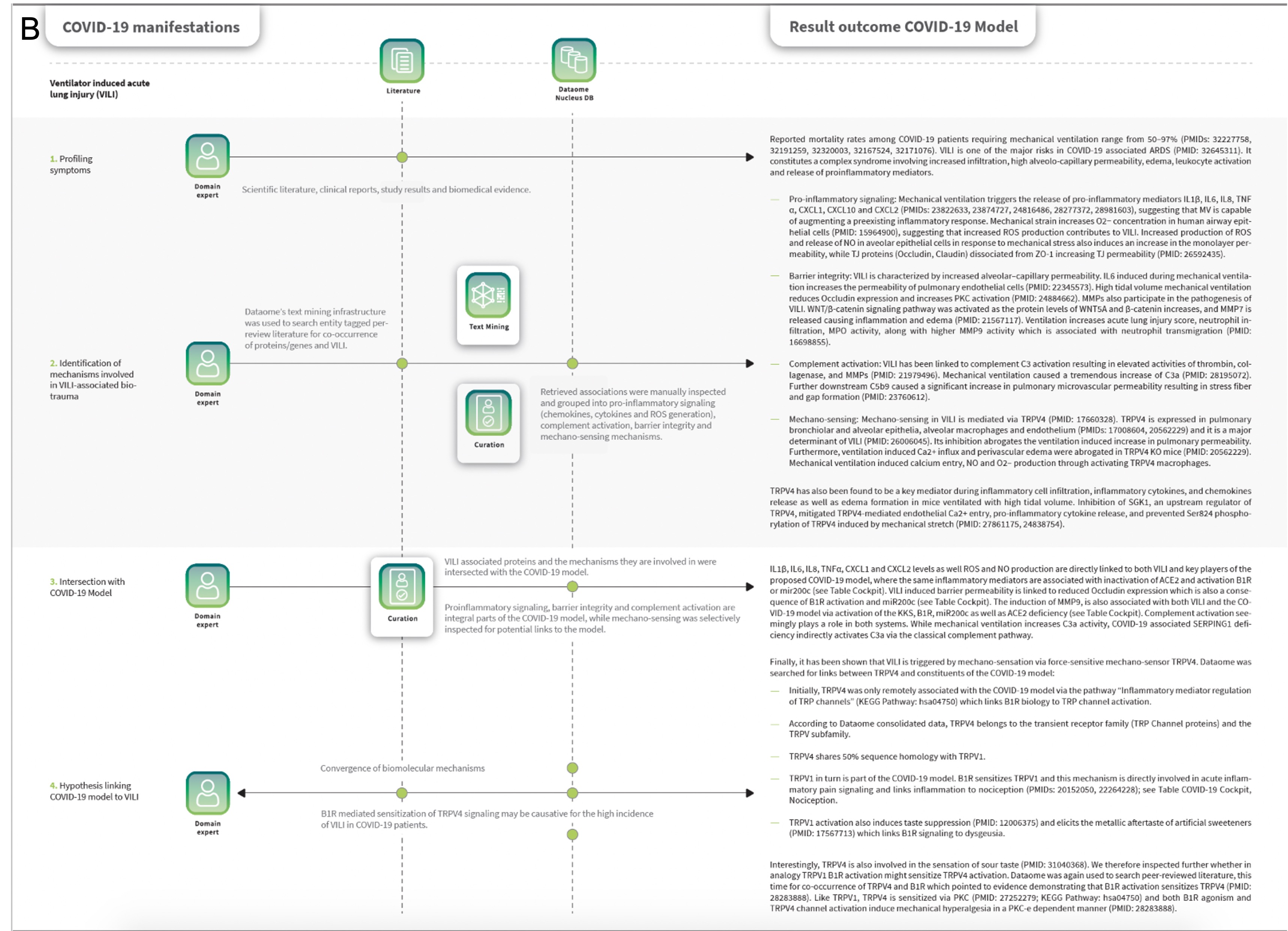

Supplement: Supplementary file 7 [file Image4.jpg]

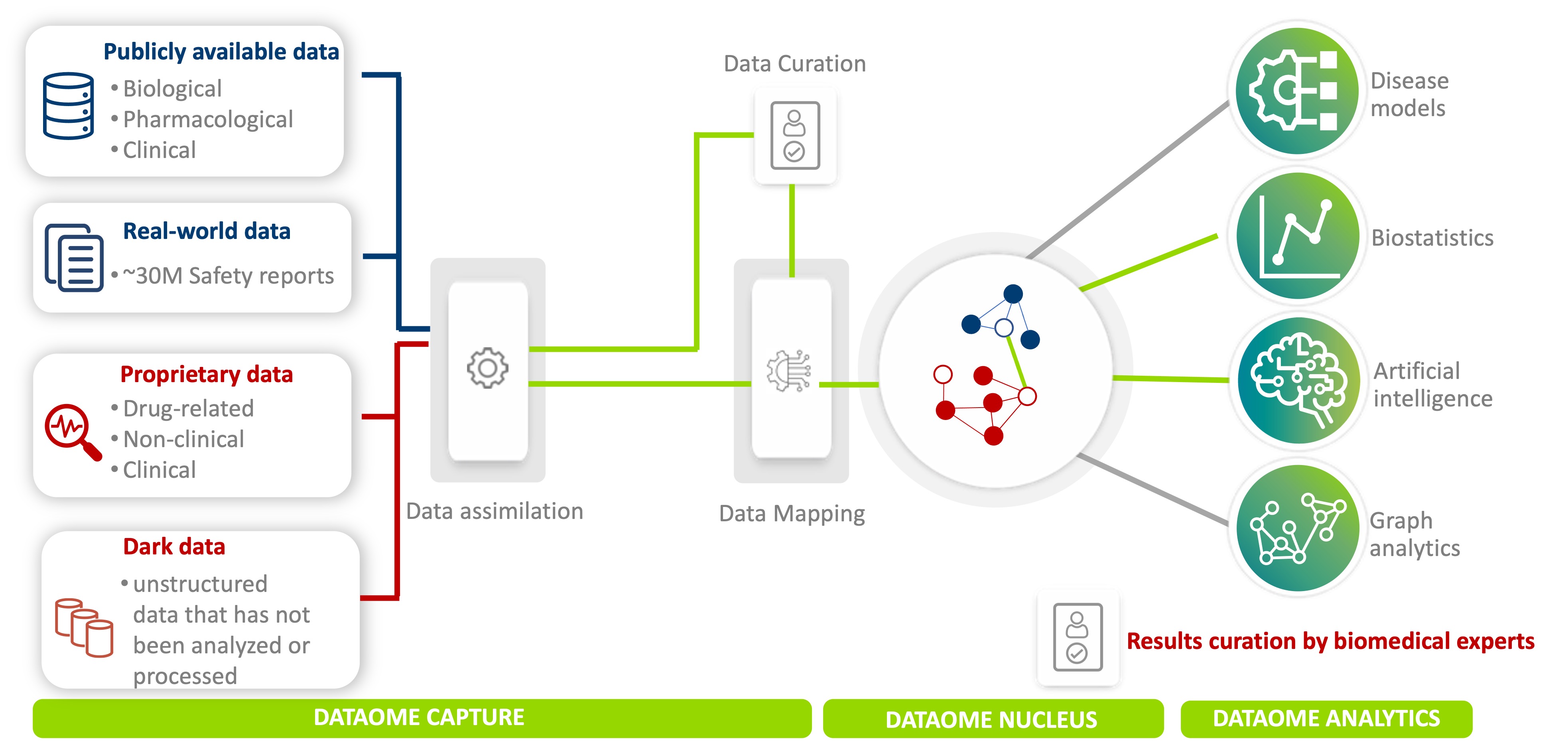

Supplement: Supplementary file 9 [file Image1.jpg]
