# Supplementary material for: The COVID-19 explorer—An integrated, whole patient knowledge model of COVID-19 disease
Source: Front Mol Med. 2022 Dec 22;2:1035215. doi: 10.3389/fmmed.2022.1035215 (PMC11285624; doi:10.3389/fmmed.2022.1035215)
Supplement: Supplementary file 5 [file Table1.docx]

**Supplement Table 1**

**Table 1:** **The COVID-19 Cockpit.**

Summary of the reported COVID-19 clinical phenotypes, pathogenic mechanisms and the relation to the COVID-19 disease model for each of the SARS-CoV-2 perturbed eight key mechanisms. A link to the COVID-19 Explorer is provided at the beginning of each section.

| **COVID-19 Cockpit** | | | |
| --- | --- | --- | --- |
| **Exocytosis** (Supplement Fig. 12, 3.3.1)  (https://covid19.molecularhealth.com/t/submodels/55.html) | | | |
| **COVID-19 Phenotype** | **Molecular Pathology** | | **Relation to Model and Findings** |
| **Neurological Dysfunction**  Cognitive deficits equivalent to >10 years ageing**^105, 106^** | - Dysregulation of the frontotemporal **SNARE** interactome is a cause of age-related cognitive decline^107^. - Global cognitive decline is associated with reduced SNARE complex levels (**Syntaxin1**, **SNAP25**, **VAMP**) and early synapse dysfunction independent of synapse loss^108, 109^. | | - Synaptic impairment due to **B1R**/**miR200c** induced repression of **Syntaxin1**. - Virus-independent propagation of symptomatology due to microvesicle (MV) based disease mechanism. |
| **Anosmia / Ageusia**  Changes in smell and taste as well as complete anosmia and ageusia are prominent and specific symptoms of COVID-19^110-113^. | - The synaptic release machinery of olfactory sensory neurons is centered on the **Syntaxin1** dependent **SNARE** complex^114^. - Taste cell synapses use the classical **Syntaxin1** **SNARE** machinery for neurotransmitter release in circumvallate taste buds^115^. | | - Olfactory sensory neurons express neither **ACE2** nor **TMPRSS2**. - Epithelial support cells, cells in the nasal epithelium, stem cells, epithelial cells of oral mucosa, in fungiform and circumvallate papillae express both of these genes^12, 14, 116^. - MVs carrying **miR200c** are transferred from infected cells to sensory cells upon excess des-Arg-kinin (DAK) and **B1R** activation may lead to suppression of **Syntaxin1**. |
| **Silent Hypoxemia**  Severe hypoxemia associated with near normal respiratory system compliance^117^. | - Similar to congenital central hypo-ventilation syndrome (CCHS), a life-threatening disorder with impaired ventilatory response to hypoxia caused by carotid body (CB) dysfunction. - CCHS patients show a significant decrease of dopaminergic vesicles in oxygen sensor cells of CBs^118^. - Sensor cells of the CB utilize the exocytotic apparatus and its components **SNAP25** and **Syntaxin1** for signaling^119^. | | - CB sensor cells are centrally exposed to circulating virus particles. - **B1R**/**miR200c** mediated downregulation of **Syntaxin1** may induce impairment of the exocytotic machinery in CB sensor cells causing silent hypoxemia. |
| **New-onset Diabetes**  New-onset diabetes and severe metabolic complications of preexisting diabetes (e.g. ketoacidosis and hyperosmolarity) have been observed in COVID-19 patients^120-122^. | - SERPING1 is down-regulated, while KNG1 is upregulated in T2D^79^ - Pancreatic islet β-cells release insulin via exocytosis of insulin secretory granules. - Exocytosis is mediated by the SNARE complex (**Syntaxin1**, **SNAP25** and VAMP)^123, 124^. - **Syntaxin1** plays a key role in Insulin granule exocytosis and replenishment^123^. - **ACE2** **deficiency** has been directly linked to defects in insulin secretion^125^. - **B1R** as well as **CPN1**, the enzyme that converts bradykinin (BK) into the **B1R** ligand **DABK**^126, 127^. **B1R** antagonism and blockade of **CPN1**^127-129^. - **B1R** sensitizes **PKCε**^130^ and B1R activation causes translocation of **PKCε**^131^ while **PKCε** contributes to lipid-induced insulin resistance^132^ - **B1R** in adipose tissue controls the response to diet-induced obesity, its deletion protects from diet-induced obesity and improves insulin sensitivity^133^. - **miR200c** is biomarker of insulin resistance in obesity^134^. - **miR200c** diminishes insulin production by inducing pancreatic β-cell damage^135^. - Suppression of **miR200c** improves β-cell function in patients with T2D and restores endothelial function in diabetic mice^136^. - **miR200c** down-regulates **IRS1^137^**. In diabetes, **IRS1** down-regulation is associated with insulin resistance^138^. - **miR200c** also targets **PGC1A**^139^, **PGC1A** controls the hepatic ratio of **IRS1** and IRS2 and in type 2 diabetes, low **PGC1A** is associated with insulin resistance^140^, in addition **TRPV4** (which is sensitized by **B1R** activation^130^) negatively regulates the **PGC1A** expression^141^ and **TRPV4** antagonists reduce high‐fat diet‐induced obesity, insulin resistance, diabetic nephropathy, retinopathy and neuropathy^141-144^. It has been demonstrated that **TRPV4** activity is mediated via the **B1R**-**PKCε** axis^130^. - **SERPING1** is down-regulated, while **KNG1** is upregulated in T2D^79^. | | - **ACE2** deficiency and **B1R**/**miR200c** mediated downregulation of **Syntaxin1** in β-cells may lead to the induction of new-onset diabetes which can persist after virus clearance. - In addition **insulin resistance** may be induced by **B1R** mediated sensitization of **PKCε** and **miR200c** mediated downregulation of **IRS1. miR200c** also down-regulates **PCG1A**, which is associated with insulin resistance. PCG1A is also downmodulated by **TRPV4,**which in turn is sensitized by **B1R** activity. |
| **Thick Mucus**  Mucous secretions of COVID-19 patients are reminiscent of those in cystic fibrosis (CF) patients^145^. | - CF is caused by mutations that disrupt the **CFTR** gene^146^. - **CFTR** is known to interact with **Syntaxin1**, chloride channel **CLC3**, and **aquaporins** to form the porosome complex^147^. - The process of secretion via the **porosome** is similar to exocytosis: a pore is formed through transient fusion of a secretory vesicle at the porosome base via **SNARE** proteins, resulting in a fusion pore^148^. | | - B1R/miR200c mediated downregulation of Syntaxin1 in lung epithelium may therefore mimic the molecular pathology of CF. |
| **Senescence** (Supplement Fig. 13)  (https://covid19.molecularhealth.com/t/submodels/46.html) | | | |
| **COVID-19 Phenotype** | **Molecular Pathology** | | **Relation to Model and Findings** |
| **Objective muscle weakness**  Muscle weakness represents one of the most typical symptoms of COVID-19^149^. | - **ACE2** is expressed in muscle cells^13^. - **ACE2** deletion leads to early manifestation of aging-associated muscle weakness along with increased expression of **p16INK4a**^150, 151^. - Induction of senescence via de-repression of **p16INK4a** in muscle stem cells is responsible for age-related decline of the regeneration capacity of muscle tissue^152^. - Activation of the **p16INK4a** senescence pathway during aging breaks muscle homeostasis and causes degenerative muscle disease^153^. - In **ACE2** knockout mice **p16INK4a** is upregulated in skeletal muscles^154^. | | - Virus induced down-regulation of **ACE2** may induce objective muscle weakness through induction of a senescence pathway. - **P16INK4a** is a universal senescence marker and accumulates with age and inflammation^155^. - **BMI1** is a transcriptional repressor of **p16INK4a**^156^. - **BMI1** is a direct target of **miR200c**^157^. - **miR200c** has been shown to repress **BMI1**^158^ |
| **Lymphopenia**  T-cell senescence and lymphopenia has been reported in patients with COVID-19 pneumonia^159^. | - **BMI1**-depleted mice show defects in self-renewal of hematopoietic stem cells. - Overexpression of **BMI1** via repression of **p16INK4a** enables stem cells overcome senescence^160-162^. - **BMI1** regulates lymphoid specification by preventing B-cell lineage commitment. **BMI1** depletion in T-cells leads to T-to-B cell conversion^162^. | | - Virus induced overactivation of the **ACE2/DAK/B1R** axis via **miR200c** mediated downregulation of **BMI1** may contribute to the development of lymphopenia. |
| **Tissue repair deficiency**  The differentiation of mesenchymal stem cells (MSCs) into type-II alveolar epithelial cells is critical for re-epithelization and recovery in ARDS^163^.  Cellular senescence is a mediator of COVID-19 severity^164^. | - Endothelial progenitor cells (EPCs) and MSCs augment tissue repair. Due to their immunomodulatory properties MSCs were extensively used in regenerative medicine^165, 166^. - MSCs were recently used to treat COVID-19^167^. - In MSCs, knockdown of **BMI1** reduces self-renewal by upregulation of **p16INK4a**^168^. - **BMI1** induces immunosuppressive properties and senescence-control mechanisms in human MSCs by directly suppressing **DUSP1**^169^. - **p16INK4a** triggers senescence in EPCs. The onset is accelerated by administration of **Kininogen**^170^. - **ACE2** priming of MSCs and EPCs enhances their therapeutic efficacy^171, 172^. - Activation of **SIRT1** supports MSC mediated repair. This effect is abolished by **ACE2** inhibiton^173^. | | - Virus-induced overactivation of the **ACE2/DAK/B1R** axis via **miR200c** mediated downregulation of **BMI1** may contribute to stem cell senescence. |
| **Alopecia**  One of the striking long-term symptoms of COVID-19 survivors is hair loss**^174, 175^** | - Dermal Papilla Cells (DPCs) play a key role in normal hair growth and are considered as a reservoir of multipotent stem cells^176^. - Balding DPCs were linked to premature senescence as they lose proliferative capacity, express senescence markers and show decreased expression of **BMI1** together with upregulation of **p16INK4a**^177^. | | - The mechanism through which **B1R** overactivation contributes to **p16INK4a** expression may provide a direct link between our model and the observed alopecia in COVID-19 patients. |
| **Inflammatory Signaling** (Supplement Fig. 9)  (https://covid19.molecularhealth.com/t/submodels/55.html) | | | |
| **COVID-19 Phenotype** | **Molecular Pathology** | | **Relation to Model and Findings** |
| **Cytokine/Chemokine Syndromes**  (Hyperinflammation)  COVID-19 is characterized by immune cell infiltration and cytokine storm^178^. | - **ACE2** is anti-inflammatory:   **ACE2** activation reduces levels of **IL6**, **IL8**, **IL2**, and **CCL2**, **TNFα**, **IL1β** via downmodulation of **NFκB**, **MAPK**, **JAK**/**STAT** and **TGFβ** pathways and increases anti-inflammatory **IL10**^179-184^. Phosphorylation of **ACE2** by **AMPK** increases its anti-inflammatory activity. - **B1R** activation mediates the pro-inflammatory effect of **ACE2** downregulation^36^.  **B1R** induces chemokines **CXCL1**, **CXCL2**, **CXCL5**^35, 36, 185^, release of **MMP9** and **MPO** via **MAPK**^186^  and increase of **phosphatidylinositol** hydrolysis, **arachidonic acid** and **eicosanoid**^187, 188^. - **miR200c** induces **IL6** and **TNFα** ^61^. Knockdown of **miR200c** represses **TGFβ**, **TNFα**, and **IL1α** via inhibiting **NFκB** and **Smad2** activation^189^. | | - Downregulation of **ACE2** leads to over-activation of **B1R** and induction of **miR200c** leading to an increase in pro-inflammatory and decrease in anti-inflammatory cytokines. - The increase in chemokines induces neutrophil infiltration, activation and degranulation. |
| **Oxidative Stress Syndromes**  COVID-19 is associated with Reactive Oxygen Species (ROS) inflammation^190^ (e.g., ARDS). | - **miR200c** is induced by oxidative stress and highly elevated in plasma from pneumonia patients^55, 56^. **miR200c** represses antioxidant proteins **eNOS**, **catalase**, **HO1** and **SOD2**^55, 58^. - **B1R** activation induces **iNOS**. | | - **B1R** and **miR200c** lead to a decrease in antioxidant enzymes and increase in **ROS** formation. - In virus-induced lung injury concurrent downregulation of **eNOS** and upregulation of **iNOS** drive **ROS** production^191^. - **ROS** lead to ARDS progression and endothelial/epithelial barrier dysfunction^59, 192, 193^. |
| **Neurogenic Inflammation**  COVID-19 is associated with neurogenic inflammation^194^. | - **B1R** activation induces the release of **SubstanceP** and **CGRP**, leading to leukocyte recruitment^35, 185^. - **B1R** induces **TRPV1**^195^, a mediator of neurogenic inflammation^196^. - **ROS** activate **TRPV1** leading to overproduction of **SubstanceP** ^197^. | | - Morbidity and severity of COVID-19 are linked to the **TRPV1** expressing neuronal system in the lungs^198^. - Respiratory viral infections can upregulate **TRPV1** receptors^199^. - **CGRP** released from the **TRPV1** nerve endings downregulates innate immune responses^200^. - Neurogenic inflammation is involved in the molecular pathology of Migraine^201^. Migraine-like headache and light-sensitivity may occur in COVID-19^202^. |
| **Nociception** (Supplement Fig. 10)  (https://covid19.molecularhealth.com/t/submodels/47.html) | | | |
| **COVID-19 Phenotype** | **Molecular Pathology** | | **Relation to Model and Findings** |
| **Cough, Myalgia, Arthralgia and inflammatory pain**  Cough, myalgia and arthralgia are common symptoms of COVID-19^203^. | - **B1R** via activation of **TRPV1** is directly involved in acute inflammatory pain signaling, linking inflammation to nociception^204, 205^. - **TRPV1** activation elicits the cough reflex^206, 207^. - **B1R** activation mediates inflammatory muscle pain^208^. It triggers release of **NO**, **SubstanceP** and **CGRP**, key mediators of myalgia^209^ and diabetic hyperalgesia^210^. - **DABK** aggravates hyperalgesia, which is reversed by **B1R** antagonism^211^. **B1R** antagonism also reverses diabetic hyperalgesia by reducing **TRPV1** expression^195^. - **TRPV1** expression depends on **PKC** mediated phosphorylation^212^, consistent with the finding that in nociception **B1R** activates **PKC**^213^. - **TRPV1** mediates arthralgia^214^ and kinin-induced pain signaling in cutaneous nociceptors, cardiac afferents, jejunal afferents and tracheobronchial afferents^215-218^. - **TRPV4** (which is  sensitized by **B1R**^130^) mediates mechanical hyperalgesia via the **B1R**-**PKCε** axis^130^. | | - **B1R** activation is directly linked to nociception and inflammatory pain. It is co-expressed with **CGRP** and **TRPV1** on sensory C^219, 220^ and A-fibers^221^. |
| **Dysgeusia**  Dysgeusia is a common COVID-19 symptom^222^. | - **B1R** is expressed in the sensory nervous system^223^  and peripheral sensory ganglia^224^. - **B1R** triggers release of **CGRP** and **SubstanceP** from trigeminal afferents, which terminate within taste buds, impacts taste-cell sensitivity and shapes taste signals^225^. - In peripheral terminals of the glossopharyngeal nerves **CGRP** release reduces taste signals before their transmission to the gustatory sensory fibers and the brain^226^. - **TRPV1** activation directly induces taste suppression^120^ and elicits the metallic aftertaste of artificial sweeteners^227^. - Metallic taste sensation was reported by COVID-19 patients^228^. | | - **B1R** activation by inducing **TRPV1** is directly linked to neurogenic signaling inducing dysgeusia. |
| **Ventilator induced acute lung injury** (VILI)  Reported mortality rates among COVID-19 patients requiring mechanical ventilation range from 50–97%^76, 91, 229-231^. VILI is one of the major risk factors^232^. | - VILI is triggered by mechano-sensation^233^. - The mechano-sensor **TRPV4**, shares 50% sequence homology with **TRPV1**. - **TRPV4** is involved in sour-taste sensing^234^, and plays a critical role in VILI^235^. - **TRPV4** is expressed in pulmonary bronchiolar and alveolar epithelia, alveolar macrophages and endothelium^236, 237^. - As **TRPV1**, **TRPV4** is sensitized via **PKC**^238^  and **B1R** agonism and **TRPV4** channels induce mechanical hyperalgesia in a **PKC**-dependent manner^130^. | | - **B1R** activation via mechanosensory **TRPV4** may induce VILI. - VILI is also associated with downregulation of TJ protein **Occludin. Occludin** expression is regulated by **miR200c** and **B1R** (see barrier permeability) |
| **Coagulation**  (https://covid19.molecularhealth.com/t/submodels/50.html) | | | |
| **COVID-19 Phenotype** | **Molecular Pathology** | | **Relation to Model and Findings** |
| **Thrombo-embolic** **events** and **disseminated intravascular coagulopathy** (DIC)  Severe COVID-19 is often associated with thrombo-embolic events and DIC^239^. Abnormal coagulation is associated with poor prognosis and non‐survivors show higher levels of D‐dimer and fibrin degradation products^240^. | - **B1R** plays an important role in mediating organ damage and lethal thrombosis in septic shock^241^. - Increased expression of **B1R** predisposes platelets to thrombus formation^241^. - **B1R** activation results in sustained elevation of **intracellular Ca^2+^**^46^, which is essential for platelet activation in thrombosis^242^. - **B1R** inhibition like **Kininogen** deficiency protects from thrombosis^243^. Kininogen deficiency also protects from vascular injury^244^  and ischemic neurodegeneration by reducing thrombosis^243^. - **Fibrin degradation products** resulting from thrombotic events increase **DABK** generation^245^. - **ACE2** attenuates thrombus formation^246^  likely by eliminating **DAKs.** Low **ACE2/ACE** ratios observed in acute pulmonary embolism^247^. - SARS-CoV induced downregulation of **SERPING1** directly induces the coagulation cascade^248^ - SARS-CoV-2 infection stimulates formation of extracellular neutrophils traps (NETosis) which contributes to COVID-19 associted thrombosis^249^. - Non-lytic NETosis involves neutrophil degranulation and triggers a process in which MPO synergizes with neutrophil elastase in decondensing chromatin to form the neutrophil extracellular traps^250^. - NET bound histones mediate NET-dependent cytotoxicity^251^. - Histones and MMP9 contribute to NET-mediated barrier disruption^252, 253^. - Inhibition of C1 is able to dose-dependently inhibit NET formation^254^. - Anti-histone properties of C1-Inhibitor SERPING1 protects against histone mediated lung injury^255^. - SARS-Cov2 induced downregulation od SERPING1 could contribute to COVID-19 associated NETosis. - B1R activation leads to degranulation of neutrophils and release of MMP9 and MPO^186^. - B1R activation may contribute to NETosis onset and sustain. | | - While downregulation of **SERPING1** directly activates the coagulation cascade, **ACE2** deficiency and **B1R** activation play a direct role in platelet activation. - Downregulation of SERPING1 and activation of B1R could directly contribute to NETosis formation and NET dependent cytotoxicity and thrombosis. |
| **Barrier Permeability** (Supplement Fig. 11)  (https://covid19.molecularhealth.com/t/submodels/56.html) | | | |
| **COVID-19 Phenotype** | **Molecular Pathology** | | **Relation to Model and Findings** |
| **Acute Respiratory Distress Syndrome** (ARDS)  One of the most common causes of hospital admission and death in patients with COVID-19 is ARDS characterized by acute lung inflammation and increased-permeability pulmonary edema^256^. Increased barrier permeability in alveolar edema, ARDS and acute lung injury (ALI) result from loss of tight junctions (TJ) permselectivity^257^, which coincides with upregulation of inflammatory cytokines. | - **B1R** stimulation results in the loss of **Occludin** TJs and an increase of vascular permeability^258^. In neuroinflammation **B1R** contributes to the loss of blood-brain-barrier integrity^259^. - Neutrophils engage the **KKS** to open the endothelial barrier in acute inflammation^260^. - Activation of **B1R** induces expression and secretion of **MMP9** and **MMP2**^261^. Active **MMP2** and **MMP9** degrade components of the alveolar basement membrane^262, 263^, non-matrix components such as integrins^264, 265^, and intercellular targets such as E-cadherin^266, 267^. - **B1R** blockade reduces edema formation in  ARDS^268^, acute ischemic stroke^269^, traumatic brain injury^259^  and multiple sclerosis^270^. - **ACE2** deficiency is associated with increased **MMP9** levels in myocardial infarction^271^. - miR200c represses Occludin^272^. Antago-miR200c inhibits MMP9 and increases Occludin^273^.      - **TMPRSS2** cleaves and activates **PAR2**^274^. In airways **PAR2** activation increases lung vascular and epithelial permeability and pulmonary edema^275^. - Activation of **SIRT1** by Resveratrol maintains the epithelial barrier by increasing the expression of TJ proteins **ZO1**, **Occludin** and **Claudin1**^276^. **SIRT1** negatively regulates **MMP9**, and reduction of **SIRT1** levels through oxidative stress confers an increase in **MMP9**^277^. - Activation of **FAS** increases barrier permeability and decreases the expression of **Occludin** and **ZO1** in the alveolar-capillary membrane and in alveolar epithelium^278^. - **FAS** is highly induced in response to **miR200c** overexpression^61^. At the same time **miR200c** represses **FAP1**, a negative regulator of **FAS**^279^. - **EZH2**-knockdown is accompanied by upregulation of **Occludin** and **Claudins**^280^. - **TRPV4** activation (which is sensitized by **B1R**,^130^) leads to increased permeability of the alveolar septal barrier and pulmonary edema^236, 281^, while TRPV4 inhibition may reduce pulmonary edema in heart failure patients^282, 283^. **TRPV4** induces reversible epithelial cell permeability^284^, while **TRPV4** antagonism counteracts the diabetes-like effects on endothelial permeability^143^. In a model of cerebral artery occlusion **TRPV4** inhibition attenuated the loss of TJ proteins **Occludin** and **ZO-1**^28^**^5^**. | | - **ACE2** downregulation, **B1R** activation and **miR200c** expression increase barrier permeability by impairing TJs and inducing enzymes degrading the extracellular matrix. - The effect **SIRT1** and **EZH2**, on barrier integrity are consistent with their regulatory roles in the ACE2-DAK-B1R axis. - Down-regulation of TJ proteins is observed in other **COVID-19** **associated pathologies** involving epithelial barrier defects such as in **kidney injury**^286^ or **Kawasaki disease,** where decreased ZO-1 levels are associated with intestinal barrier dysfunction^287^. - In **VILI** and inflammatory lung diseases predisposing patients to severe lung failures, **Occludin** and **ZO1** are reduced in alveolar epithelia cells^288^. Up-regulation of **Occludin** ameliorates **VILI**^289^  and **unfractionated heparin** attenuates ALI by upregulating **Claudin**, **ZO1** and **Occludin**^290^. - **COVID-19** patients with respiratory failure show increased **MMP9**^291^. **MMP9** degrades the base membrane. Levels of **MMP9** are elevated and predictive in ALI/ARDS^292, 293^. **MMP9** also impairs and actively degrades components of the BBB, leading to the development of **cerebral edema**^294, 295^. In **CKD**, **MMP9**^296^. - **Aging** exacerbates ALI-induced changes of the epithelial barrier, lung function, and inflammation. ALI in old mice is associated with high **MMP9** and significantly reduced **Occludin** levels^297^. This is consistent with the role of members of the ACE2-DAK-B1R signaling axis in ageing. - **TRPV4**, which is sensitized by **B1R** activity, may contribute to increased barrier permeability and edema. |
| **Fibrogenic Signaling**  (https://covid19.molecularhealth.com/t/submodels/54.html) | | | |
| **COVID-19 Phenotype** | **Molecular Pathology** | | **Relation to Model and Findings** |
| **ARDS-associated fibrosis**  COVID-19 patients develop ARDS, that is characterized by rapid onset fibrosis^76^. | - **ACE2** is protective but downregulated in human lung **fibrosis**^298, 299^. - **ACE2** inhibits signaling pathways of tissue fibrosis in models of atherosclerosis, cerebral ischemia, obesity, chronic kidney disease, liver diseases and asthma^300^. **ACE2** deficiency increases cardiac mortality and adverse remodeling^301^. - Pharmacological activation of **ACE2** prevents lung fibrosis^302^, inhibits cardiac fibrosis and decreases cardiac collagen^303^. - **DAKD** engagement of **B1R** stimulates **Collagen** synthesis^304^ . Inhibition of **B1R** reduces renal fibrosis in obstructive nephropathy^305^ and ameliorates glomerulonephritis^306^ . Deletion of **B1R** reduces renal fibrosis^307^  and attenuates cardiac fibrosis^308^. - **Kallikrein** and **TMPRSS2**, via activation of **EGFR** contribute to fibrosis formation. **EGFR** signaling has been linked to increased fibrosis after **SARS-CoV** infection^309^ . In vascular smooth muscles, **Kallikrein** stimulates ADAM17 activity via a **PAR1/2** receptor-dependent mechanism, leading to the release of the endogenous ADAM17 substrates, amphiregulin and TNFα and MMP-dependent transactivation of **EGFR**^310^. **EGFR** contributes to the onset of pulmonary fibrosis^311^. Activation of **PAR2** by **Kallikrein** induces **EGFR** transactivation^310^  and in airways **PAR2** contributes to pulmonary fibrosis^312^. **PAR2** dependent transactivation of **EGFR** has been shown to contribute to renal fibrosis^313^. - **TMPRSS2** is an activator of **PAR2**^274^. - **miR200c** drives the development of fibrosis. It increases expression of **collagen** and **fibroblast growth factor**^86^. **miR200c** is aberrantly expressed in fibrosis of lung, liver, kidney, peritoneum and skin and serum levels are an early diagnostic biomarker of lung fibrosis^85^ . - Stressed cardiomyocytes release MVs highly enriched in **miR200c**, inducing the spread of fibrotic events^314, 315^. - In prostate cancer, **miR200c** induces expression of **EGR1**^61^. **SARS-CoV** induces **EGR1** dependent activation of **TGFβ** leading to a profibrotic response^316^. Targeting of **EGR1** ameliorates fibrosis in the lung^317^. - Overexpression of **miR200c** enhances **mortalin**, activity^318^. **Mortalin** deficiency suppresses fibrosis^319^ . - Hepatitis C Virus induced **miR200c** promotes fibrosis^86^. - **SIRT1** attenuates **TGFβ** mediated lung fibrosis^320^  and protects against pulmonary fibrosis^321^ **. TGFβ** downregulates **ACE2** expression in a **SIRT1**-dependent manner^322^. Pharmacological activation of **SIRT1** attenuates fibrosis^323^  and **Metformin**, an enhancer of **SIRT1** and **AMPK** expression^324^  reverses lung fibrosis. **AMPK** activity is reduced in fibrotic regions^325^ . - **TRPV4** is sensitized by **B1R**^130^. **TRPV4** plays a significant role in fibrosis of multiple organ systems including myocardial fibrosis, cystic fibrosis, pulmonary fibrosis, hepatic fibrosis and pancreatic fibrosis, reviewed in^326^. | | - **ACE2** downregulation, **B1R** activation and **miR200c** expression promote fibrosis. - The effects **Kallikrein**, **TMPRSS2**, **SIRT1 and** **AMPK**, on fibrogenic signaling are consistent with their respective roles in the context of the **ACE2**/**DAK**/**B1R** **axis**. - **TRPV4** which is sensitized by **B1R** activity, is mechanistically associated with fibrosis in multiple organs. |
| **Autoimmunity**  (https://covid19.molecularhealth.com/t/submodels/63.html) | | | |
| **COVID-19 Phenotype** | | **Molecular Pathology** | **Relation to Model and Findings** |
| **Autoimmune diseases (e.g., Kawasaki disease)**  COVID-19 precedes the appearance of various autoimmune diseases^327^. Autoimmune hemolytic anemia, immune thrombocytopenic purpura, Guillain-Barré syndrome have each been reported in patients with COVID-19^328^. Clinical manifestations resembling Kawasaki Disease n children is associated with a new phenotype of autoimmunity^329^. Antineutrophilic autoantibodies (ANCAs) have been identified in cases of COVID-19 associated vasculitis and glomerulonephritis^330^  and an increased risk of thrombotic events appears to be associated with the presence of antiphospholipid autoantibodies^331-334-292^. Disease severity and poor clinical outcomes are correlated with intense activation of extrafollicular B-cell responses^335^. The immunological landscape associated with this effector B cell mobilization in COVID-19 is similar to the one observed in patients with active autoimmune processes. Similar to systemic lupus erythematosus (SLE), antibodies generated through this process could be pathogenic, on the basis of ^336, 337^. | | - **B1R** is involved in the development of autoimmune diseases such as multiple sclerosis^338^, Crohn’s disease, ulcerative colitis, and inflammatory bowel disease^339, 340^. - **B1R** is upregulated on T-cells from peripheral blood of patients with multiple sclerosis^270, 341^. - Leukocyte **B1R** plays a critical role in the pathogenesis of **ANCA** glomerulonephritis^287^. - **B1R** blockade suppresses systemic autoimmunity in lupus nephritis^342^. - **B1R** is expressed on dendritic cells^343^. **B1R** blockade ameliorates systemic immunity by silencing dendritic cells and helper T cells. - Inhibition of **B1R** protects from autoimmune CNS disease^270^. - **miR200c** is elevated in primary immune thrombocytopenia^344^. - Autoimmunity is tightly connected to stem cell senescence and **immune-senescence**. As described above, the **ACE2**/**DAK**/**B1R** axis via dysregulation of **p16INK4a** might drive stem cell senescence. SLE patients show enhanced senescence of MSCs^183^, which is induced by upregulation of **p16INK4a**^345^. | - **B1R** activation and **miR200c** are associated with autoimmunity. - Immune-senescence induced via the **ACE2**/**DAK**/**B1R** axis might be involved in the autoimmunity phenotype. |
