# Supplementary material for: The COVID-19 explorer—An integrated, whole patient knowledge model of COVID-19 disease
Source: Front Mol Med. 2022 Dec 22;2:1035215. doi: 10.3389/fmmed.2022.1035215 (PMC11285624; doi:10.3389/fmmed.2022.1035215)
Supplement: Supplementary file 10 [file DataSheet1.docx]

**Supplement File 1**

**The COVID-19 Explorer – Patient-level insights into the molecular symptomatology of COVID-19 disease**

**Stephan Brock ^5^, Theodoros G. Soldatos ^5^, David B. Jackson ^5^, Francesca Diella ^5^, Klaus Hornischer ^5^, Anne Schäfer ^5^, Simon P. Hoerstrup ^1,2, *^, Maximilian Y. Emmert ^1,2,3,4, *^**

*^1^Institute for Regenerative Medicine, University of Zurich, Zurich, Switzerland*

*^2^Wyss Zurich, University of Zurich and ETH Zurich, Zurich, Switzerland*

*^3^Department of Cardiothoracic and Vascular Surgery, German Heart Institute Berlin, Berlin, Germany*

*^4^Department of Cardiovascular Surgery, Charité Universitätsmedizin Berlin, Berlin, Germany*

*^5^Molecular Health GmbH, 69115 Heidelberg, Germany*

***Correspondence:**
**Corresponding Authors**
[**maximilian.emmert@irem.uzh.ch**](mailto:maximilian.emmert@irem.uzh.ch)
[**simon.hoerstrup@irem.uzh.ch**](mailto:simon.hoerstrup@irem.uzh.ch)

Address for Correspondence:

Professor Maximilian Y. Emmert, MD, PhD

Institute for Regenerative Medicine (IREM),

Moussonstrasse 13, 8044 Zurich, Switzerland

Tel.: +41 44 634 5610

Fax : +41 44 634 56 08

E-Mail: [maximilian.emmert@irem.uzh.ch](mailto:maximilian.emmert@irem.uzh.ch)

Professor Simon P. Hoerstrup, MD, PhD

Institute for Regenerative Medicine (IREM),

Moussonstrasse 13, 8044 Zurich, Switzerland

Fax : +41 44 634 56 08

Tel.: +41 44 634 5610

E-Mail: [simon.hoerstrup@irem.uzh.ch](mailto:simon.hoerstrup@irem.uzh.ch)

**Keywords: COVID-19, SARS-CoV-2, molecular mechanisms, disease modeling, evidence-based medicine, translational research, knowledge engineering**

Table of Contents

[1 Glossary 3](#_Toc111795179)

[1.1 List of abbreviations 3](#_Toc111795180)

[1.2 HGNC symbols of listed gene names 5](#_Toc111795181)

[2 Materials and Methods 6](#_Toc111795182)

[2.1 Data Availability 6](#_Toc111795183)

[Online Availability 6](#_Toc111795184)

[3 References 9](#_Toc111795185)

# Glossary

## List of abbreviations

ACI: Augmented Clinicomics Intelligence

ACMG: American College of Medical Genetics

AD: Alzheimer’s disease

AF: Atrial fibrillation

AHFS: American Hospital Formulary Service

ALI: Acute lung injury

ANCAs: Antineutrophilic auto-antibodies

AR: Androgen receptor

ARDS: Acute respiratory distress syndrome

ATC: Anatomical Therapeutic Chemical (ATC) classification system

BBB: Blood Brain Barrier

BK: Bradykinin

CAS: Contact activation system

CCHS: Congenital central (hereditary) hypoventilation syndrome

CB: Carotid body

CF: Cystic fibrosis

CNS: Central nervous system

COPD: Chronic Obstructive Pulmonary Disease

COVID-19: Coronavirus disease 2019

DAK: des-Arg-kinins (i.e., DABK and/or DAKD)

DABK: des-Arg^9^-kinins

DAKD: des-Arg^10^-KD

DIC: Disseminated intravascular coagulation / coagulopathy

DIZE: Diminazene aceturate

DPC: Dermal papilla cells

EHR: Electronic health records

EPC: Endothelial progenitor cell

ESMO: European Society for Medical Oncology

ETL: Extraction, Transformation, and Loading

FAERS: FDA’s Adverse Event Reporting System (USA)

FDA: Food and Drug Administration (USA)

GBS: Guillain–Barré syndrome

GPML: GenMAPP Pathway Markup Language

GWAS: Genome-wide association study

HGNC: HUGO Gene Nomenclature Committee

HK: High molecular weight kininogen

HPO: Human Phenotype Ontology

HUGO: Human Genome Organisation

ICD: International Classification of Disease

ICTRP: International Clinical Trials Registry Platform

ISG: Interferon stimulated gene

ISO: International Organization for Standardization

KD: Kallidin

KWD: Kawasaki disease

KEGG: Kyoto Encyclopedia of Genes and Genomes

KKS: Kallikrein Kinin system

LPS: Lipopolysaccharide

MedDRA: Medical Dictionary for Regulatory Activities

MESH: Medical Subject Headings

MH: Molecular Health GmbH

MI: Myocardial infarction

MSC: Mesenchymal stem cell

MV: Microvesicle

NCCN: National Comprehensive Cancer Network

NLM: National Library of Medicine (USA)

NLP: Natural language processing

PAH: Pulmonary arterial hypertension

PH: Pulmonary hypertension

PK or PreKK: Prekallikrein

RAS: Renin-angiotensin system

RCT: Randomized clinical trials

ROS: Reactive Oxygen Species

RWD: Real world data

SARS-CoV: Severe Acute Respiratory Syndrome CoronaVirus

SLE: Systemic lupus erythematosus

SVG: Scalable Vector Graphics

T2D: Type 2 Diabetes

TDM: Text-/Data-Mining

TJ: Tight junction

UCSC: University of California-Santa Cruz

UMLS: Unified Medical Language System

VILI: Ventilator induced (acute) lung injury

WHO: World Health Organization

## HGNC symbols of listed gene names

| **Gene name(s) in publication text** | **HGNC** | |
| --- | --- | --- |
| (main article and supplementary material) | **Approved symbol** | **ID** |
| *AP1 (or c-Jun)* | JUN | 6204 |
| *APP* | XPNPEPL1; XPNPEP2 | 12822; 12823 |
| *AT1R* | AGTR1 | 336 |
| *AT2R* | AGTR2 | 338 |
| *B1R* | BDKRB1 | 1029 |
| *B2R* | BDKRB2 | 1030 |
| *Beclin-1* | BECN1 | 1034 |
| *Claudin1* | CLDN1 | 2032 |
| *CLC3* | CLCN3 | 2021 |
| *CypA* | PPIA | 9253 |
| *eNOS* | NOS3 | 7876 |
| *FAB1* | PIKFYVE | 23785 |
| *FXII* | F12 | 3530 |
| *gC1qR* | C1QBP | 1243 |
| *IFNγ* | IFNG | 5438 |
| *IL1α* | IL1A | 5991 |
| *IL1β* | IL1B | 5992 |
| *IL8* | CXCL8 | 6025 |
| *iNOS* | NOS2 | 7873 |
| *Kininogen (and derivatives BK, DAK)* | KNG1 | 6383 |
| *KK (or preKK or PK)* | KLKB1 | 6371 |
| *KLK2* | KLK2 | 6363 |
| *Mas1R* | MAS1 | 6899 |
| *Mortalin* | HSPA9 | 5244 |
| *NEP* | MME | 7154 |
| *NFκB* | NFKB1 | 7794 |
| *Occludin* | OCLN | 8104 |
| *Oct-1* | **POU2F1** | 9212 |
| *p16INK4a* | CDKN2A | 1787 |
| *PAR2* | F2RL1 | 3538 |
| *PEA3* | ETV4 | 3493 |
| *Pit-1a* | POU1F1 | 9210 |
| *POP, PEP* | PREP | 9358 |
| *Syntaxin1* | STX1A | 11433 |
| *TMEM27* | CLTRN | 29437 |
| *TNFa* | TNF | 11892 |
| *XCR* | XCR1 | 1625 |
| *ZO1* | TJP1 | 11827 |
| *HO1* | HMOX1 | 5013 |

# Materials and Methods

## Data Availability

## Online Availability

As the COVID-19 Explorer and the associated knowledge resource represents one of the main parts of our interactive paper, all data, methods and code/software associated with the atlas of whole patient symptomatology and the molecular pathways associated with these physiological effects can be downloaded within a dedicated download section of the COVID-19 Explorer interface, accessible at:

<https://covid19.molecularhealth.com/basemodel>

<https://covid19.molecularhealth.com/submodels?type=disease>

<https://covid19.molecularhealth.com/submodels?type=functional>

**(Login Information: Username: review, Password: E3cYN4pK).**

Furthermore, other raw, un-curated data pertaining to any aspect of this submission, will also be made available upon reasonable request. In addition, the following supplementary files and folders are provided:

**Supplementary Data File 3**

The supplementary file **``Submodels.csv``** contains summaries of all submodels along with associated symptoms, genes, organs, and the underlying references as Pubmed Unique Identifiers (PMIDs)

**Supplementary Data File 4**

The supplementary Zip Folder **``submodels_svg``** contains SVGs files of all submodels.

**Supplementary Data File 5**

The supplementary Zip Folder **``submodels_csv``** contains CSV files containing all binary interactions of the submodels.

In this context, it is also important to emphasize that the development of the COVID-19 Explorer was enabled by our DATAOME platform. The DATAOME is an expansive biomedical data and analytics infrastructure that has been developed for over a decade by our collaborators at Molecular Health Inc., in Heidelberg, Germany

# Figures


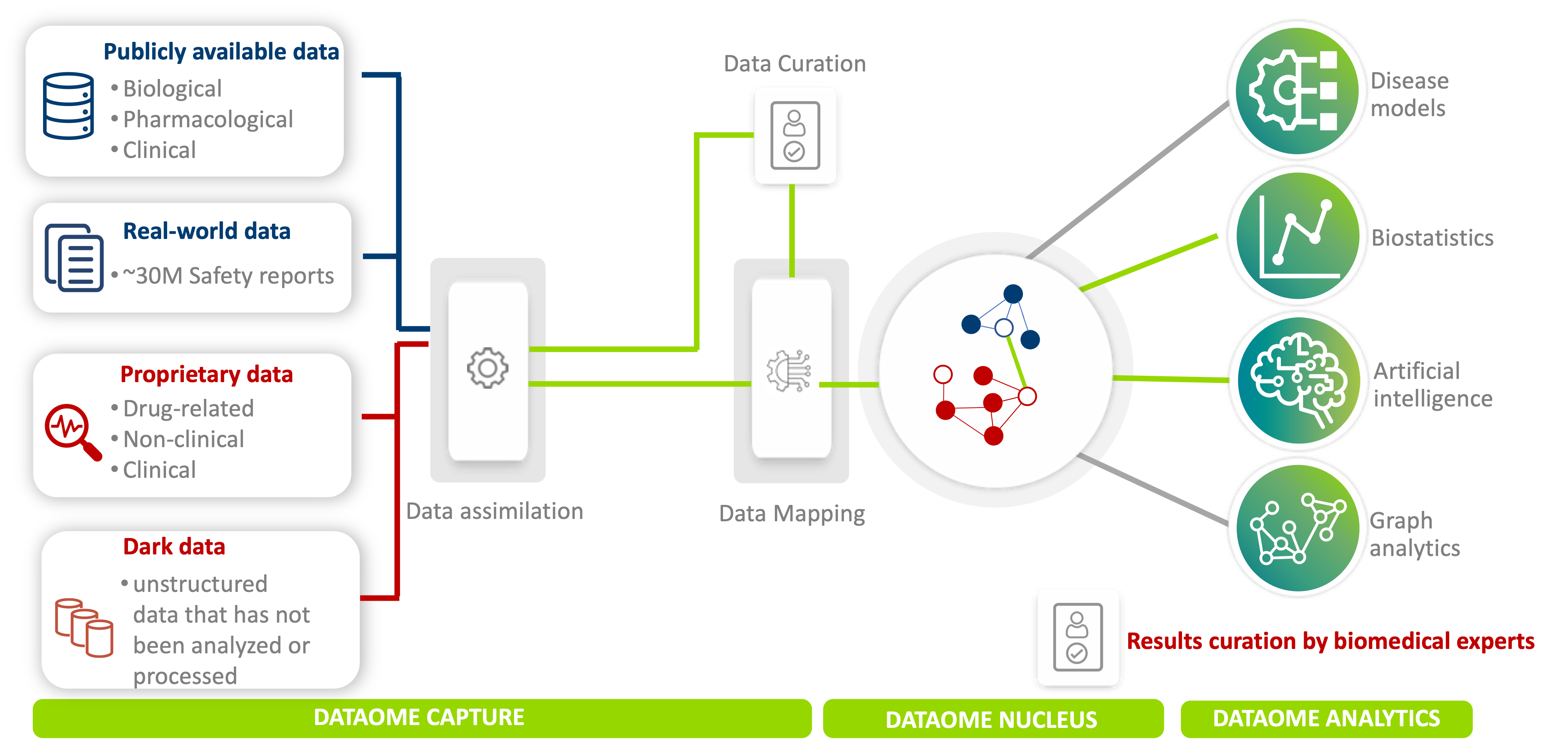


**Supplement Figure 1: Schematic overview of the Dataome technology.**

The Dataome technology provides structured integration of clinical (e.g. Real World Data (RWD), Randomized Control Trials (RCT), Spontaneous Reporting System (SRS) and Electronic Health Record (EHR)) together with molecular data and knowledge. Through the *Data Capture* module, data is integrated (using text data mining (TDM) and automated Extraction, Transformation and Loading (ETL) processes) and normalized before integration into the *Data Nucleus*, using a transformation pipeline. This integrated data then serves as the clinico-molecular knowledgebase upon which decision support technologies and discovery applications are built (*Data Analytics*) providing opportunities regarding next-generation predictive models, precision medicine biomedical analytics and actionable insights.


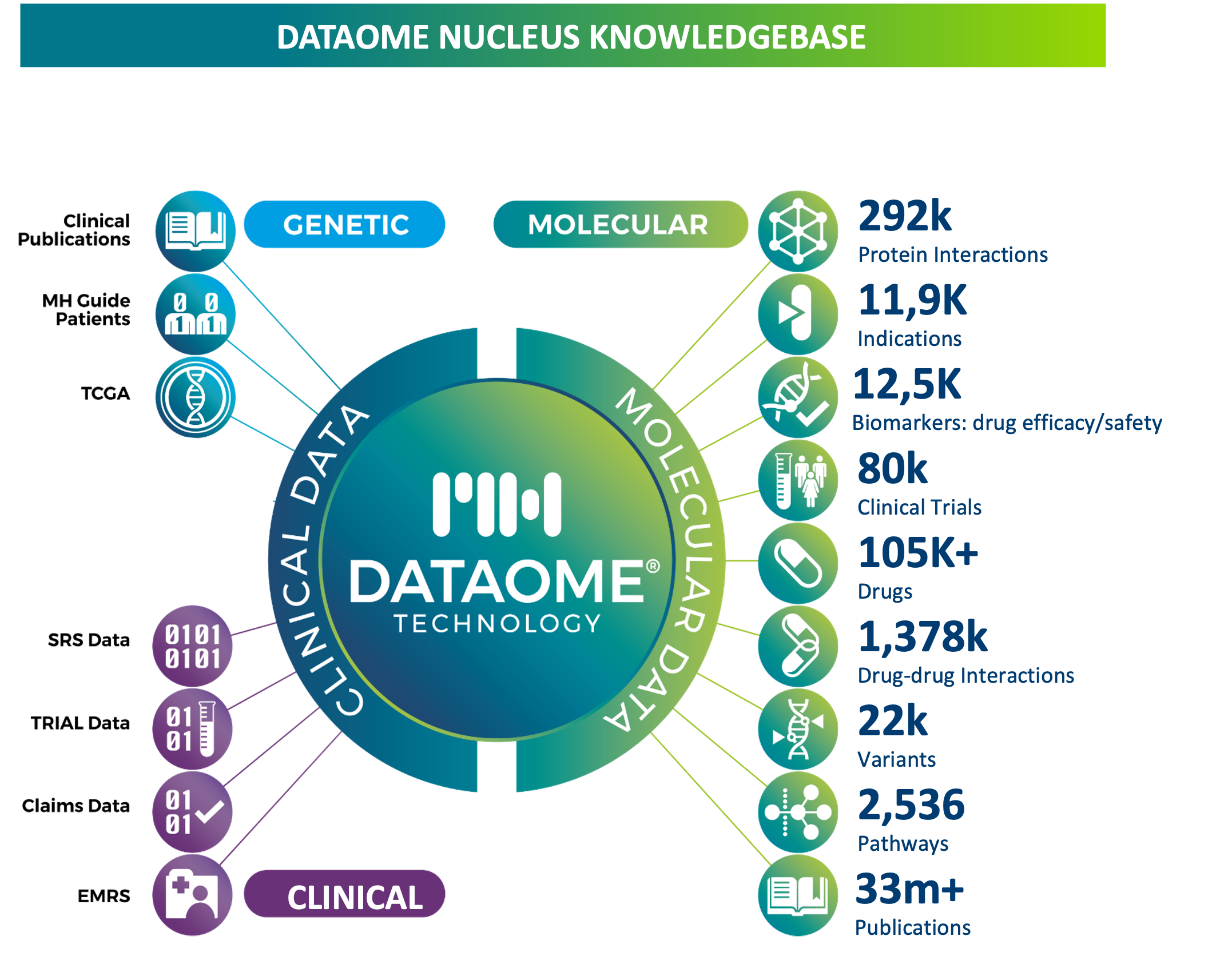


**Supplement Figure 2: Schematic overview of the Dataome Nucleus Knowledgebase and associated datatypes.**

The Dataome Nucleus integrates clinical and molecular information from public, proprietary and commercially available data sources. The datawarehouse includes clinical outcomes data for millions of individual patients (e.g. from spontaneous reporting systems), structured and referenced to globally available molecular data and knowledge. The data is constantöy optimized with respect to seven key parameters 1) quality, 2) quantity, 3) Diversity, 4) Novelty, 5) Actuality 6) Security and 7) Utility, through a combination of automated and manual curation process. It also includes several proprietary databases for clinical outcomes, drugs, pathways and genomic variants.


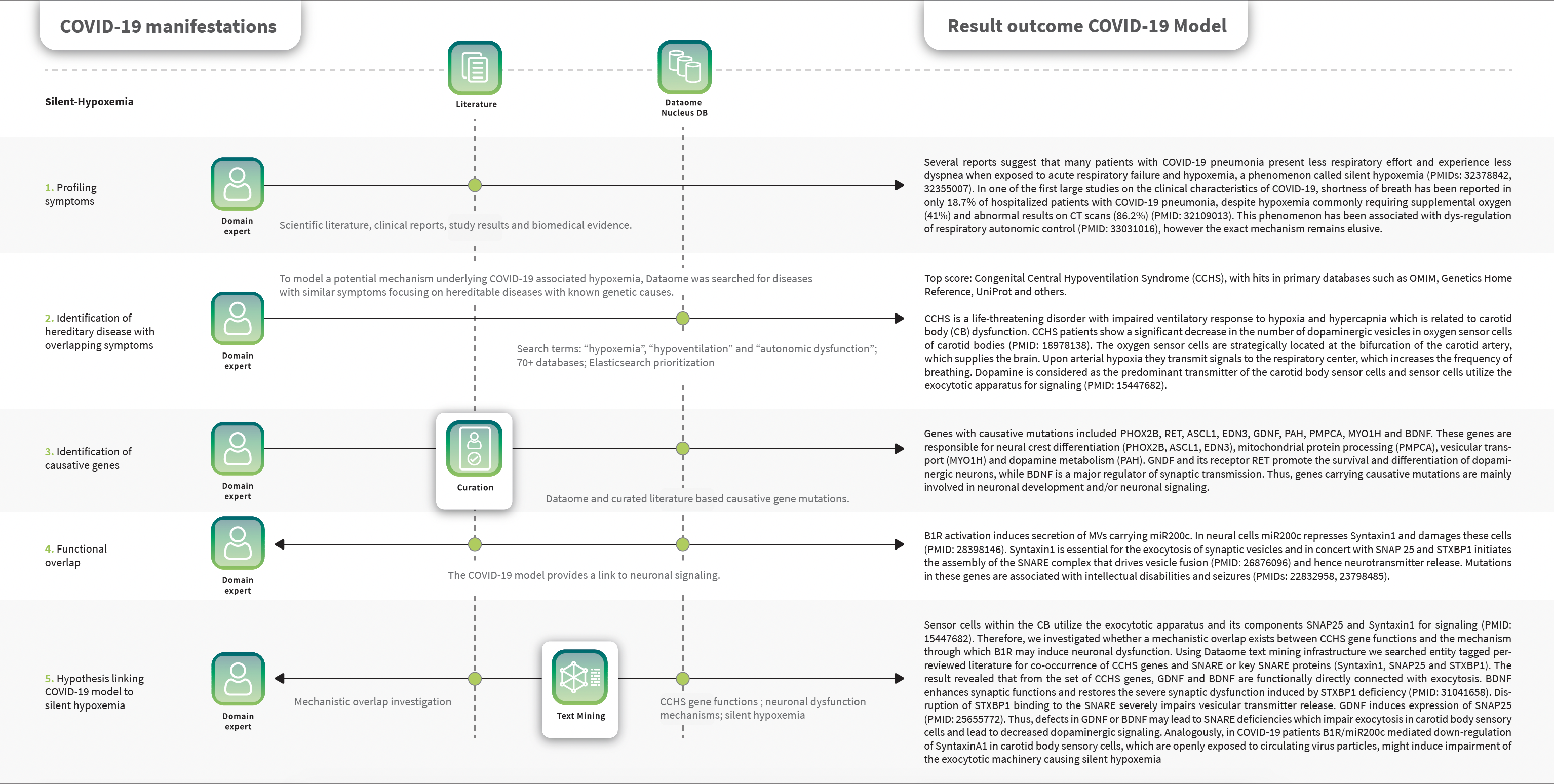


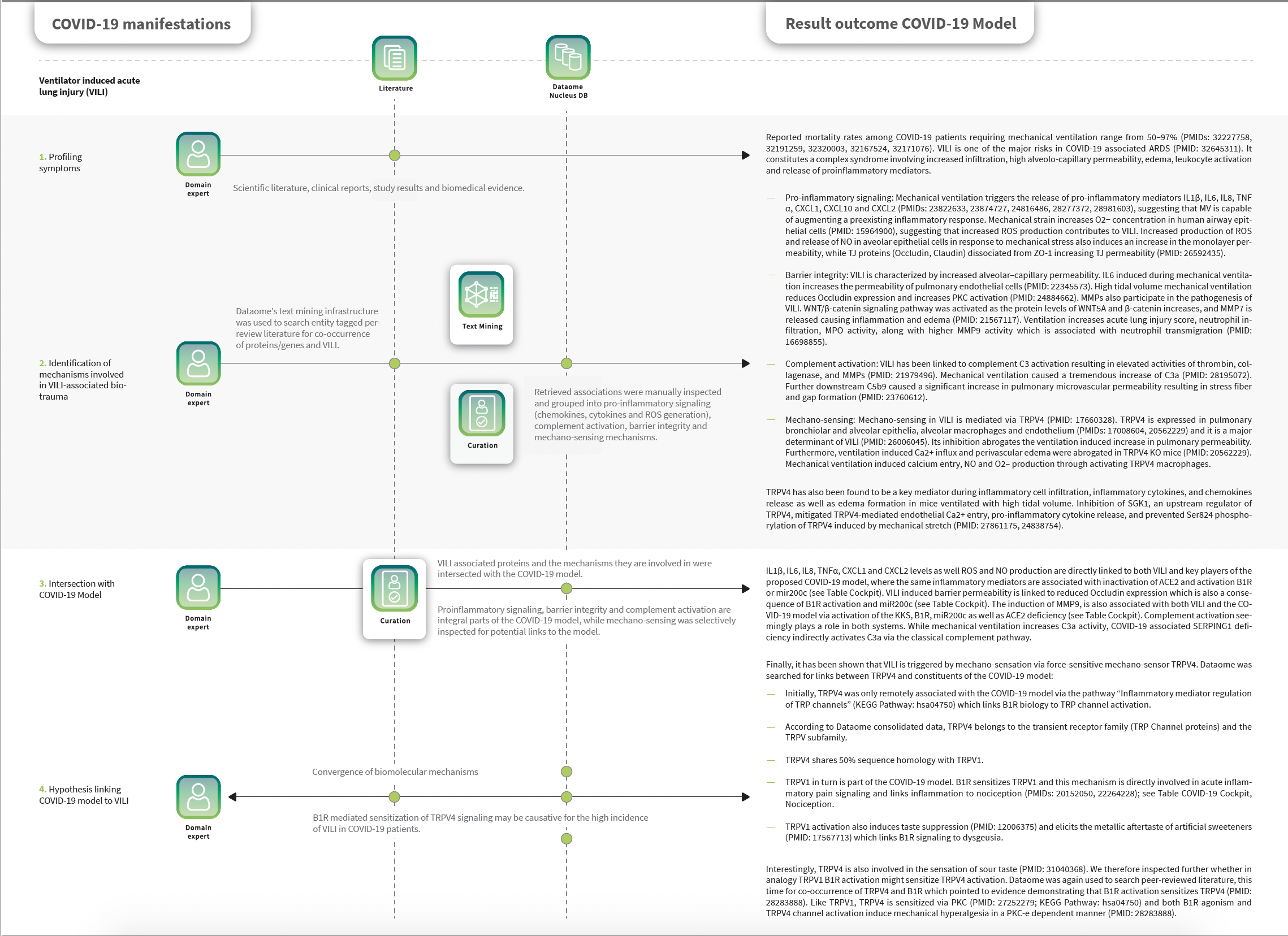


**Supplement Figure 3: Detailed example of the workflows of two case-study examples (Silent hypoxemia and Ventilator-induced Lung Injury (VILI)), corresponding to figure 3 in the main text.** The top diagram illustrates the key steps towards the identification of functional link with Congenital Central Hypoventilation Syndrome (CCHS) genes via neuronal signaling/dysfunction mechanisms. The lower diagram describes how we linked *B1R* mediated sensitization of *TRPV4* signaling to the incidence of ventilator induced acute lung injury (VILI) in COVID-19 patients (for more details see also Supplementary files 2 (Table 1).
